# Preparation and purification of organic samples for selenium isotope studies

**DOI:** 10.1371/journal.pone.0193826

**Published:** 2018-03-06

**Authors:** Helena Banning, Monika Stelling, Stephan König, Ronny Schoenberg, Thomas Neumann

**Affiliations:** 1 Karlsruhe Institute of Technology, Institute of Applied Geosciences, Adenauerring 20 b, Karlsruhe, Germany; 2 University of Tuebingen, Department of Geosciences, Wilhelmstraße 56, Tuebingen, Germany; 3 Technical University Berlin, Institute of Applied Geosciences, Applied Geochemistry Group, Berlin, Germany; INRA, FRANCE

## Abstract

Selenium (Se) is an important micronutrient but also a strong toxin with a narrow tolerance range for many organisms. As such, a globally heterogeneous Se distribution in soils is responsible for various disease patterns (i.e. Se excess and deficiency) and environmental problems, whereby plants play a key role for the Se entrance into the biosphere. Selenium isotope variations were proved to be a powerful tracer for redox processes and are therefore promising for the exploration of the species dependent Se metabolism in plants and the Se cycling within the Critical Zone. Plant cultivation setups enable systematic controlled investigations, but samples derived from them–plant tissue and phytoagar–are particularly challenging and require specific preparation and purification steps to ensure precise and valid Se isotope analytics performed with HG-MC-ICP-MS. In this study, different methods for the entire process from solid tissue preparation to Se isotope measurements were tested, optimized and validated. A particular microwave digestion procedure for plant tissue and a vacuum filtration method for phytoagar led to full Se recoveries, whereby unfavorable organic residues were reduced to a minimum. Three purification methods predominantly described in the literature were systematically tested with pure Se solution, high concentrated multi-element standard solution as well as plant and phytoagar as target matrices. All these methods efficiently remove critical matrix elements, but differ in Se recovery and organic residues. Validation tests doping Se-free plant material and phytoagar with a reference material of known Se isotope composition revealed the high impact of organic residues on the accuracy of MC-ICP-MS measurements. Only the purification method with no detectable organic residues, hydride generation and trapping, results in valid mass bias correction for plant samples with an average deviation to true δ^82/76^Se values of 0.2 ‰ and a reproducibility (2 SD) of ± 0.2 ‰. For phytoagar this test yields a higher deviation of 1.1 ‰ from the true value and a 2 SD of ± 0.1 ‰. The application of the developed methods to cultivated plants shows sufficient accuracy and precision and is a promising approach to resolve plant internal Se isotope fractionations, for which respective δ^82/76^Se values of +2.3 to +3.5 ‰ for selenate and +1.2 to +1.9 ‰ for selenite were obtained.

## Introduction

Selenium is an ultra-trace element but an essential nutrient for the human body at low concentration levels, while at somewhat higher concentration levels it becomes toxic [1]. This narrow margin between Se deficiency and toxicity, as well as the fact that plants are the main Se source for humans and animals, make it increasingly important to optimize a healthy Se dosage in global food supplies such as rice and wheat. At present times, however, worldwide Se uptake by cultivated plants is strongly heterogeneous, with crops in many areas showing Se deficiency, e.g. in China [2], Southern Africa [3–4] and Northern Europe [5], or even toxic levels, e.g. in Punjab [6] and California [7]. A fundamental problem with maintaining a healthy Se uptake in cultivated plants is that pathways of this trace element from soil to plants and within the plants are not sufficiently understood.

The determination of Se isotope ratios is a powerful tool in the environmental sciences and geosciences, which was already applied to shales, hydrothermal and volcanic rock samples, marine and terrestrial sediments, soils as well as microbial and fungi cultures [8–26]. For the majority of these studies Se isotopes were reported to be a very good redox tracer due to its high and species dependent isotope fractionation in redox reactions. This sensitivity makes Se isotope signatures a promising device in the exploration of the Se cycle in and related to plants, which underlies varying pathways depending on Se source concentration, redox species, soil and water properties as well as land use. In this context, reductive Se transformations play a crucial role [27].

However, only few spot data exist on Se isotope signatures in higher plants. [10] describe a Δ^82/76^Se_plant-water_ value (i.e. δ^82/76^Se_plant_—δ^82/76^Se_water_) between plant and soil pore-water of -1.5 ‰ in a wetland, meaning a slight depletion of heavy isotopes in the plant. [24] investigated Se isotope signatures in wheat crops irrigated with Se rich water and found contrary Δ^82/76^Se_plant-water_ values of +2.5 ‰ and +3.2 ‰, which corresponds to a significant enrichment of heavy isotopes in the plants. Those data indicate that on the one hand Se isotope signatures are sensitive to plant related processes and on the other hand may reflect plant or ecosystem specific Se cycling. However, in these studies in situ framework conditions were too complex to trace and differentiate particular processes.

That is why we decided to apply a minimum parameter plant cultivation approach, which includes a closed system with rice plants in an artificial growth medium, phytoagar, and as far as possible excludes inaccuracies by natural soil, water and weather fluctuations as well as organisms other than the plants themselves. Challenging consequence of this simplification approach is the sample material, low amounts of plant tissue and the artificial growth medium, for which particular preparation and purification methods were essential to ensure accurate and valid Se isotope determinations. Therefore, this study aims to (1) develop, implement and validate preparation methods to bring plant tissue and phytoagar in a liquid and organic free form, (2) find and evaluate methods to purify samples from matrix elements to avoid interferences in Se isotope analytics, (3) validate the comprehensive method and define analytical precisions for each sample matrix and (4) find out if this precision is sufficient to trace plant internal Se transformation processes.

## Materials and methods

### Experimental setup of minimum parameter plant cultivation

The minimum parameter plant cultivation is a modified approach after [28]. Sterilized nutrient-free phytoagar (Duchefa powder, 0.4 wt.%) was poured into sterilized Magenta boxes and doped homogeneously with sodium selenate and sodium selenite in concentrations of 500 and 1000 μg L^-1^ respectively. 16 seeds per box of *Oryza sativa japonica*, sterilized with ethanol and sodium hypochlorite, were seeded into the phytoagar in a sterile environment. Magenta boxes were closed and placed into a climate chamber at 28°C (day, 8 h), 22°C (night, 16 h) and 70% humidity for 16 days.

To test the applicability and significance of the preparation and purification methods presented in sections 2.2 and 2.3, root and shoot samples of rice plants supplied with 500 μg L^-1^ and 1000 μg L^-1^ each selenate and selenite were analyzed for δ^82/76^Se. The Se isotope fractionation of root-shoot transfer was calculated in order to find out if the methodical precision reached for plant tissue was sufficient to address plant internal processes described in section 3.4.

### Development of sample preparation methods

After cultivation, the plants were removed from phytoagar and their sizes and weights were determined. Roots and shoots were separated from each other, washed and filled into a 2 mL Eppendorf cup containing a 5 mm diameter stainless steel bead. The cups were frozen with liquid nitrogen and directly transferred into an electric mill (Tissue Lyzer, Qiagen). After milling at 30 Hz for 120 s samples were freeze-dried (Alpha 1–4 Freeze Dryer, Martin Christ) for 24 h in open cups and grinded afterwards to a homogeneous powder.

**Fig 1:**
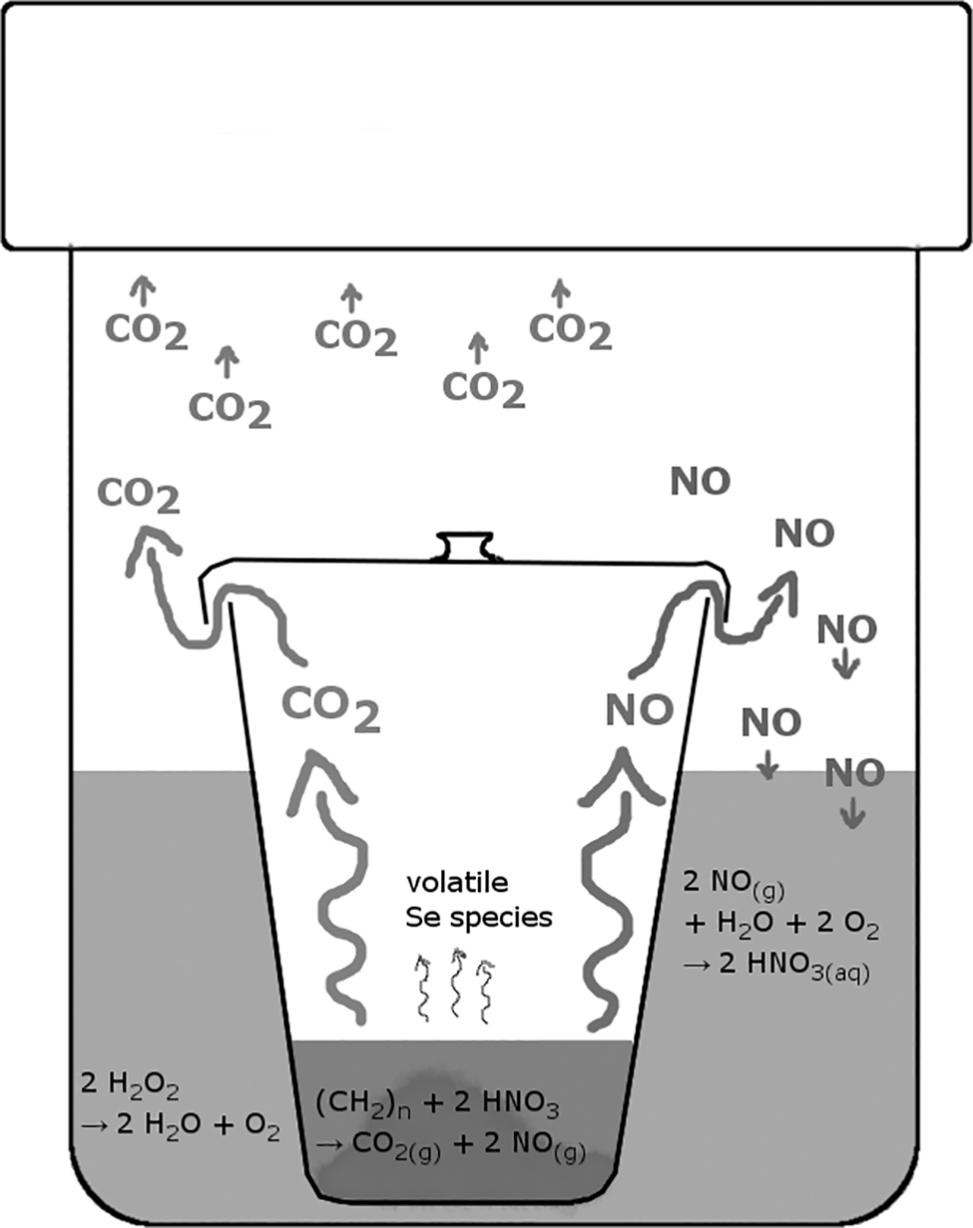
Microwave digestion method according to [30] (modified) with quartz inlay in microwave beakers allows a separation of sample chemicals, which minimizes Se losses and contamination and enhances efficiency.

For dissolution and organic destruction two digestion methods were applied and compared. The method by [29] was approved for Se concentration measurements. Accordingly, 0.1 g plant tissue was introduced in a PFA microwave beaker, mixed with 3 mL HNO_3_ (65%, VWR analytical grade, inhouse double destilled), 1 mL H_2_O_2_ (30%, Merck, suprapure grade) and 1 mL H_2_O (Millipore), treated in a microwave (START1500, MLS) at up to 210°C and cooled down for 12 h before opening. The second method was a modification after [30]. Accordingly, 0.1 g plant tissue was placed into a 30 mL quartz vessel with a loose lid on top, 2 mL HNO_3_ (65%) were added, the lid was closed and the vessel was placed into a PFA microwave beaker filled with 7 mL diluted H_2_O_2_ (8.5%) (Fig 1). The microwave beakers were tightly closed and placed into the same microwave system as described above. The samples were heated up to 240°C and cooled down for 12 h before opening. Both methods were evaluated on Se recovery and mineralization rates using a plant reference material, NIST1567a, in which Se and C_org_ concentrations were compared before (certified values and solid sample analytics) and after digestion.

The modified method after [30] was applied for phytoagar as well using 2 mL of the semi-solid material. Furthermore, it was compared to a vacuum filtration method, in which the liquid phase was partly extracted with a vacuum pump (1400 RPM; KNF Freiburg) connected to a 100 mL filter flask associated with a 120 mL Buechner funnel, and 70 mm diameter cellulose acetate filters with 0.45 μm pore width (all Roth). A full separation of liquid and solid phase was not possible, that is why we tested for Se recovery in concentration before and after extraction depending on the Se species and concentration added. Phytoagar samples doped with selenate, selenite and organic Se (selenomethionine) in concentrations of 100, 500 and 1000 μg L^-1^ were produced and treated as described. After filtration 3 mL of each HNO_3_ (65%) and H_2_O_2_ were added to the samples and the closed sample beakers were heated up to 80°C for 12 h to destroy organic compounds. Selenium recoveries and organic residuals were determined for both modification after [30] and vacuum filtration.

### Evaluation of purification methods

After dissolution and destruction of dissolved organic matter samples were purified from critical matrix elements that bear the potential to induce isobaric interferences or other analytic disturbances. Methods available in literature were neither systematically tested on purification efficiency nor implemented for organic rich samples before. Therefore the performance of three predominantly described methods, namely chromatographic anion exchange (CAE), chromatographic thiol retention (CTR) and hydride generation and trapping (HGT), were tested with pure Se solution, multi-element standard solution (Merck) containing critical elements (10 mg L^-1^ Na, Mg, Al, Ca, Cr, Fe, Co, Ni, Cu, Zn; 1 mg L^-1^ Se, As, Ge), digested Se rich plants from Punjab (India) (1 μg L^-1^ Se) as well as aliquots of plants and phytoagar digests/extracts doped with 1 μg L^-1^ Se with sample volumes of 10 mL each.

CAE makes use of selenate’s affinity to bidentate outerspheric sorption to positively charged surfaces. A commercial anion exchange resin, AG1-X8 (Bio-Rad), was applied as packing material for chromatographic separation of Se from the sample matrix [11, 15–16]. AG1-X8 powder was washed with methanol, 1M NaOH (pure) and 1M HCl (suprapure) (all Merck) and 1.2 mL of the suspended resin in chloride form was poured into a 5 mL mini-column (SpectrumLabs). The resin was activated with 6M HCl and neutralized afterwards by adding H_2_O. Prior to application, the digests or extracts of the samples were evaporated at 70°C to near dryness and taken up in 10 mL H_2_O. 100 μL 25 mM K_2_S_2_O_8_ were added and heated up at 120°C (heating plate temperature) for 60 min to fully oxidize Se to selenate [15]. These solutions were added to the column. The eluates discharged from the column–referred to as CAE *sample eluate*–were kept for analyses. Afterwards 20 mL H_2_O was added to the column to wash out soluble, but retained matrix compounds. The eluates emitted from the columns–referred to as CAE *wash eluate*–were kept for analyses as well. The Se molecules bound to the column packing were extracted by adding 6 mL HCl to exchange selenite by the more prone Cl^-^ again. These eluates–referred to as CAE *Se extract*–were kept for analyses as well. In analogy, there are *sample eluate*, *wash eluate* and *Se extract* solutions to be analyzed for the other methods tested.

CTR is based on selective Se retention by covalent thiol-Se bonds in thiol activated cellulose powder (TCP). The powder was produced according to [31] and the purification procedure was slightly modified from the same study. Accordingly, 0.1 g of TCP was filled into 5 mL minicolumns and activated by adding 2 mL each H_2_O, 6M HCl and 1M HCl. In contrast to CAE, sample solutions were evaporated at 70°C to near dryness, taken up in 4M HCl and fully reduced to Se(IV) on a hotplate at 80°C for 90 min prior to application. These solutions were diluted to 10 mL 1M HCl and added to the column. The eluates–referred to as CTR *sample eluate*–were kept for analyses. In a next step, 6M HCl was added to remove thiol prone metals such as Cu by forming soluble metal-Cl compounds. The eluates–referred to as CTR *wash eluate–*were kept for analyses. The Se molecules bound to the TCP were extracted by repeated boiling with 33% HNO_3_. The TCP-HNO_3_ suspensions were filtered and the solutions–referred to as *Se extract*–were kept for analyses.

For both methods CAE and CTR, the solutions from each step which were kept for analyses–*sample eluate*, *wash eluate* and *Se extract*–were analyzed for Se and matrix elements. The *Se extracts* from plant and phytoagar samples were analyzed for residual total organic carbon (TOC) as well. Selected *Se extracts* were analyzed for Se isotope composition.

HGT uses the characteristic of Se to form gaseous hydrides (H_2_Se), which enables the separation of Se from the matrix via gas phase trapping [13]. For this purification method, sample solutions were diluted to 4M HCl and fully reduced to Se(IV) at 80°C for 90 min. Afterwards they were cooled down and diluted to 2M HCl. Selenium hydrides were formed by adding 0.06M NaBH_4_, stabilized with 0.1M NaOH, into a gas-liquid separator (FIAS 400, Perkin Elmer). The gas phase was trapped in an alkaline peroxide solution consisting of 5 mL 1M NaOH and 1 mL 30% H_2_O_2_. Afterwards, the solution was heated up for 1 h at 80°C (heating plate temperature) to transform all Se into selenate and destroy residual H_2_O_2_. To remove the strong alkaline matrix and by-trapped non-Se hydride generating compounds, CAE was applied afterwards. *Sample* and *wash eluates* as well as *Se extracts* were analyzed on Se and matrix element contents. In the *Se extract* organic carbon and the Se isotope composition were additionally determined.

### Instrumental analytics and methods

Element concentrations were determined on a small aliquot of the digested samples using inductively coupled plasma mass spectrometry (ICP-MS) (ThermoFisher Scientific X-Series II), and Se was measured in collision cell modus. All measurement series included blanks to control memory effects as well as Se standard solutions and a reference material (CRM-TMDW (High-Purity Standards, Charleston, USA)) to monitor instrumental bias. Residual organic compounds were quantified in the solution as TOC using TOC cube (elementar). The organic carbon content of solid NIST1567a was measured with CSA (5003, Leybold-Heraeus), organic Se species in selected samples were detected using HPLC-ICP-MS with a method described by [32].

Stable Se isotope ratios have been measured on NeptunePlus multicollector inductively coupled mass spectrometer (MC-ICP-MS) (ThermoFisher Scientific) hosted at the Isotope Geochemistry Group, Department of Earth Sciences, University of Tuebingen. The samples were introduced to the instrument’s plasma using a Teledyne Cetac HGX-200 Hydride Generator with the addition of methane to the gas hydrides in order to increase the ionization efficiency of Se while decreasing polyatomic interferences such as Ar-dimers and hydrides, e.g. [33]. A detailed description of the analytical method for Se isotope measurements employed in this study is given by [34]. For mass bias correction, a Double Spike approach was used according to [35] and following [34]. This techniques offers the advantage that it is able to correct isotope fractionation occurring at sample preparation, which is essential for Se due to its sensitivity to isotope fractionation and the difficulty to recover Se quantitatively [34, 35]. An adequate amount of the Se Double Spike solution, calculated from the determined Se concentration to yield a Double Spike/sample-Se ratio of 1:1 [36], was added to the digested sample before Se purification, which is described in section 2.3. Full equilibration between sample and Double Spike requires all sample-Se to be present in inorganic form after digestion, as equilibration of inorganic Se with organically bound Se is very unlikely under prevalent conditions. To approach the availability of organically bound Se in the plant digests, the residual TOC was determined in samples of certified NIST1567a (wheat flour), which were processed according to the digestion method described here. The mineralization rate was derived from the TOC residuals in the samples, which were 30.51 (±18.91) mg L^-1^ (n = 9). On average, this corresponds to a residual organic fraction of 0.69%. We assume that organic Se molecules have comparable mineralization rates than similar organic molecules in general (without Se). Thus, a residue organic Se fraction of 0.69% equals an absolute amount of 0.6 ng Se remaining organically bound after digestion of 0.1 g plant tissue, which contains 0.11 μg Se. This amount can be regarded as low enough to not bias the overall results to a critical extent even if it is excluded from Se isotope measurements.

### Comprehensive method validation

The performed validation procedure covers the entire sample preparation to check if the preparation and purification methods applied are sufficiently effective or if they induce bias themselves. Three parameters were checked independently: (1) The presence of isobaric interferences, (2) the internal and external reproducibility as well as (3) the validity of the measured Se isotope ratios.

With the exception of Ge, the most critical interferences on Se masses are polyatomic hydride or oxide compounds. Thereby, oxygen compounds are impossible to monitor simultaneously together with the Se masses as this exceeds the mass spectrometers mass dispersion of 16% [37]. To check if the various samples were sufficiently purified, the elemental masses of each potentially critical hydride or oxide molecule were tested for signal intensity with HG-MC-ICP-MS.

The highest matrix concentrations tested are given in Table E in S1 File (*tolerance test*), which represents the maximum impurity concentrations that could be tolerated by the analytics during our measurements without detectable negative effects. Therefore, masses 58 (^58^Fe^16^O; ^58^Ni^16^O), 59 (^59^Co^16^O), 60 (^60^Ni^16^O), 62 (^62^Ni^16^O), 64 (^64^Ni^16^O; ^64^Zn^16^O), 66 (^66^Zn^16^O) and 75 (^75^As^1^H) were determined to search for potential isobaric interferences. Additionally, those masses, which tend to inhibit hydride generation, but have no direct interference potential (Cr, Mn, Fe and Cu oxides), were measured. These are masses 50, 52, 53, 54, 55, 56, 57, 63 and 65 [38]. Contents of Ge could be monitored simultaneously with the Se masses on m/z of 72 and 73 [35].

Indirect influences of preparation methods that cannot be covered by the direct analytical check were tested with a self-made standard material. Because no organic reference material certified on Se isotope composition was available, Se free target matrix, plant and phytoagar, was created in a Se free separate cultivation setup. The Se isotope standard NIST3149 was added to those samples before treating with the methods described in sections 2.2 and 2.3 in parallel approaches. Samples were measured for Se isotope composition and a δ^82/76^Se ratio corresponding to NIST3149 was expected, reflecting there was no contamination or mass bias during preparation and purification.

In order to create an internal reference material for Se isotope determinations in plant matrices and to provide a reference point for further Se isotope studies in plants, NIST1567a was repeatedly prepared with the modification of [30] (section 2.2) and HGT (section 2.3), and its Se isotope composition was determined.

## Results and discussion

### Quality of Se extraction from organic matter

Plant reference material NIST1567a, used for method validation, was certified on Se concentration (1.1 ±0.2 ppm dry wt.). Its organic carbon content was measured to be 42.8 ±0.69 wt.% (n = 6). The average Se recovery using digestion after [29] was 0.94 ±0.06 ppm (n = 31) with high blanks of 0.1–1.3 μg Se (absolute) detected in the microwave beakers of control samples without Se containing samples. Residual TOC in the digests were 335 ±37.3 mg L^-1^ (n = 9), which equals 7.83% of the initial organic carbon content. Assuming a similar fraction of total Se being available as organic compound, almost 8% would be excluded from Se isotope determinations, because organic Se will not properly equilibrate with the Double Spike-Se. A spot test for organic Se compounds confirmed our results by measuring even 16% residual organic Se in a plant sample digested according to [29]. This fraction might induce significant mass bias, making digestion after [29] less suitable for Se isotope applications.

**Fig 2:**
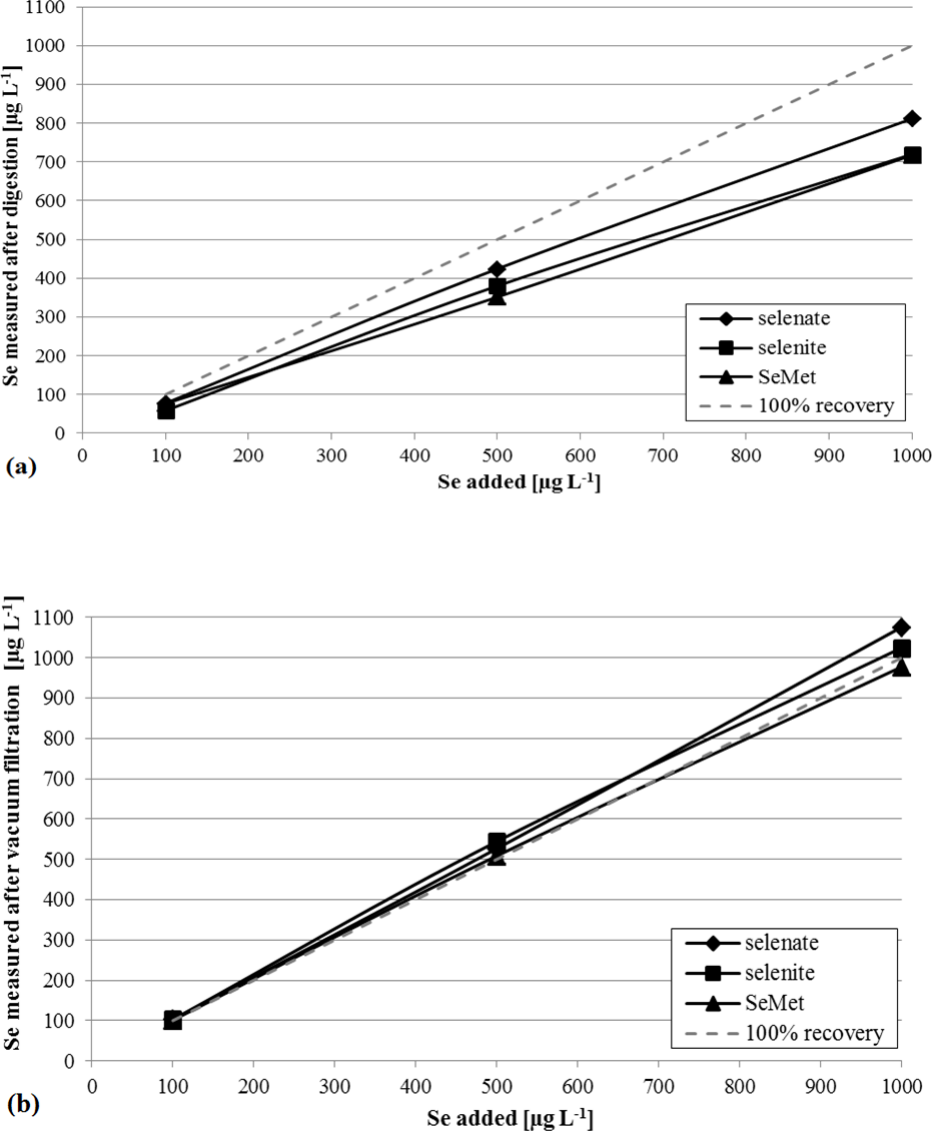
Se recoveries from phytoagar transformation into liquid form in dependence on Se concentration and species (data with external reproducibility Table A in S1 File). (a) Se recovery after digestion (after [30], average recovery 74.3 ± 5.5%), (b) Se recovery after vacuum filtration (average recovery 103 ± 2.6%).

The modified procedure after [30] resulted in a Se yield of 1.01 ±0.08 ppm (n = 9), which is within the certified range and on average 7.4% higher than digests after [29]. All Se blanks measured were <0.01 μg (absolute), probably because the quartz vessels with plain surfaces were not prone to Se retention and easier to clean than the porous PFA microwave beakers. The TOC residues were 30.5 ±18.9 mg L^-1^ (n = 9), which correspond to 0.69 ±0.42% of the initial organic carbon content. [39], who tested four state-of-the-art digestion procedures for organic material, characterized TOC residuals of <2% in digests as fully mineralized, which was only reached for one of their tested methods at 240°C. This indicates the high quality of the digestion procedure after [30] with a mineralization rate that is 91% higher than the one of [29]. A system of two separated containers likely enabled this higher rate, because it allows NO_x_ and CO_2_ to degas from the quartz vessel. Outside the inner vessel, NO_x_ is captured within the H_2_O_2_ solution and transformed to aqueous HNO_3_, which decreases the overall pressure and thereby enables higher temperatures without exceeding technical limits. Heavier volatile Se species likely remain within the inner quartz vessel and transform to the aqueous form again at cooling, which increases the Se recovery and avoids Se migration into the PFA beaker (Fig 1). Comparing both digestion methods, the modification after [30] is clearly favorable regarding Se recovery, organic matter mineralization and blank minimization. However, the method after [30] is not successfully transferable to phytoagar as illustrated in Fig 2(A). The average Se recovery in the digestion set up was only 74.3 ±5.5% with significant differences among Se species. The higher recoveries of selenate might be caused by its kinetic stability and the lower affinity for volatile emission compared to selenite and the organic Se species selenomethionine [40]. However, high Se isotope fractionation by volatilization was reported by several studies as well [8, 15, 35]. This indicates potential effects on Se isotope composition and biased results with mixed species, making this method inadequate for phytoagar treatment. Organic matter was fully destroyed by this procedure as the residual TOC was <0.9 mg L^-1^ (<0.04% of initial C_org_) in every sample. In contrast, Fig 2(B) shows that vacuum filtration effectively results in full Se recovery (103 ±2.6%) and good reproducibility with low dependencies from Se species. However, after extensive oxidation with HNO_3_-H_2_O_2_ TOC was still at 112 ± 51.5 mg L^-1^ (n = 3) (6% of initial C_org_). Longer reaction times under a certain pressure or higher temperatures might improve the mineralization rates, but involve the danger of Se losses. Therefore, with limitations, vacuum filtration seems to be a suitable phytoagar preparation method and favorable compared to digestion after [30].

### Efficiency of purification methods

#### Selenium recovery and organic residuals

Table 1 summarizes the Se recoveries for all purification methods tested, CAE, CTR and HGT, determined in the *Se extract* solutions from several matrices. For CAE, pure Se solution (1 μg) without matrix elements reproducibly resulted in full recovery, which proves the principle functionality of the method. However, high occurrence of inorganic (multi-element solution) as well as organic (Punjab plants and phytoagar) compounds severely impact the Se recovery to <50%. The low recovery of phytoagar doped with inorganic Se documents the strong influence of the organic matrix, even if not directly bound to Se, and highlights the necessity of total organic matter mineralization. Thereby full recovery of Se doped plant tissue confirms the suitability of the digestion method chosen and lets CAE appear suitable for plant sample purification. Unfortunately, CAE is sensitive to various inorganic and organic compounds and thereby not universally reliable regarding Se recovery.

**Table 1:** Se recoveries and external reproducibility tested with purification methods CAE, CTR and HGT depending on sample matrices (reference: CRM-TMDW recovery 101.3 (± 3.6) %).

method	CAE			CTR			HGT				
sample matrix	Se recovery [%]	TOC [mg L^-1^]	n	Se recovery [%]	TOC [mg L^-1^]	n	Se recovery HG* [%]	Se recovery total*** [%]	n	TOC [mg L^-1^]	n
**pure Se (Roth)**	100 ± 0.8	n/a	3	72 ± 1.1	n/a	3	82 ± 5.6	n/a	2	n/a	
MS** (Roth)	45 ± 7.0	n/a	8	79 ± 13.9	n/a	7	0.05 ± 0.01	n/a	2	n/a	
**Se rich plants (Punjab)**	40 ± 19.6	n/a	9	89 ± 18.8	n/a	7	n/a				
**Se free plants + doped Se**	99 ± 1.9	7.8 ± 1.8	3	74 ± 1.0	19.9 ± 4.7	3	87 ± 7.3	53 ± 20.1	10	<0.9	3
**Phytoagar filtrate**	49 ± 16.9	73.0 ± 32.0	3	77 ± 0.7	49.9 ± 25.4	3	92 ± 2.9	54 ± 2.3	2	<0.9	3

*hydride generation only (HG) without anion exchange step

**ICP multi-element standard containing 100 μg Na, Mg, Al, Ca, Cr, Fe, Co, Ni, Cu, Zn and 10 μg As, Ge, Se

***aliquots containing 1 μg Se

In contrast, Se recoveries of CTR ranged between 72 and 89% for all matrices applied, with generally good reproducibility and no detectable dependencies on matrix compounds. CTR seems to be applicable for many sample types to reach reliable Se recoveries on a high level.

The third method tested HGT (without CAE) reached even better Se recoveries of 82–92% for pure Se solution, plant and phytoagar samples. Organic residues appear not to influence hydride generation and trapping significantly, whereby high inorganic element concentrations do so, which is evident by multi-element standard Se recovery close to 0%, and in accordance with [36]. These authors reported an inhibition of hydride generation at high prevalence of Fe, Ni, Co, Cu and As, which might explain the low Se recovery. In case that transition metals show high concentrations in the target samples, a previous removal step (e.g. with CTR) should solve this problem. The CAE step following hydride generation, which is necessary to remove the strong alkaline NaOH matrix from the trap and ensure Se(IV) stability, lead to significant Se losses during the procedure and decreased Se recoveries to 50% (cf. section on matrix removal pathways).

In addition to Se recoveries, Table 1 shows the TOC residues in the samples after purification with CAE, CTR and HGT. In purified plant digests they were low in absolute numbers, but made a significant fraction of the digest’s TOC, namely on average 14% for CAE and 35% for CTR respectively. Dissolved organic matter was likely retained in the pore spaces of the packing materials or sticked to its charged surface and extracted afterwards. The higher value for CTR was probably caused by cellulose from TCP mobilized during the extract phase. Phytoagar extracts released 5 to 10 times more organic carbon into the purified sample than plant tissues. In relation to the initial TOC it was with 65% (CAE) and 44% (CTR) significantly higher than plant digests, too. The larger and more structured reaction surface might have caused the higher number for CAE. Additional organic destruction by HNO_3_ boiling might have further decreased TOC in CTR. In samples purified with HGT residual TOC could be detected neither in the plant digests nor in the phytoagar extracts. At hydride generation, the major fraction of the organic residues probably remained in the liquid phase. Organic hydrides, which are likely to be generated [41, 42], are not prone to be captured in a peroxide trap.

#### Matrix removal pathways and element residuals

**Fig 3:**
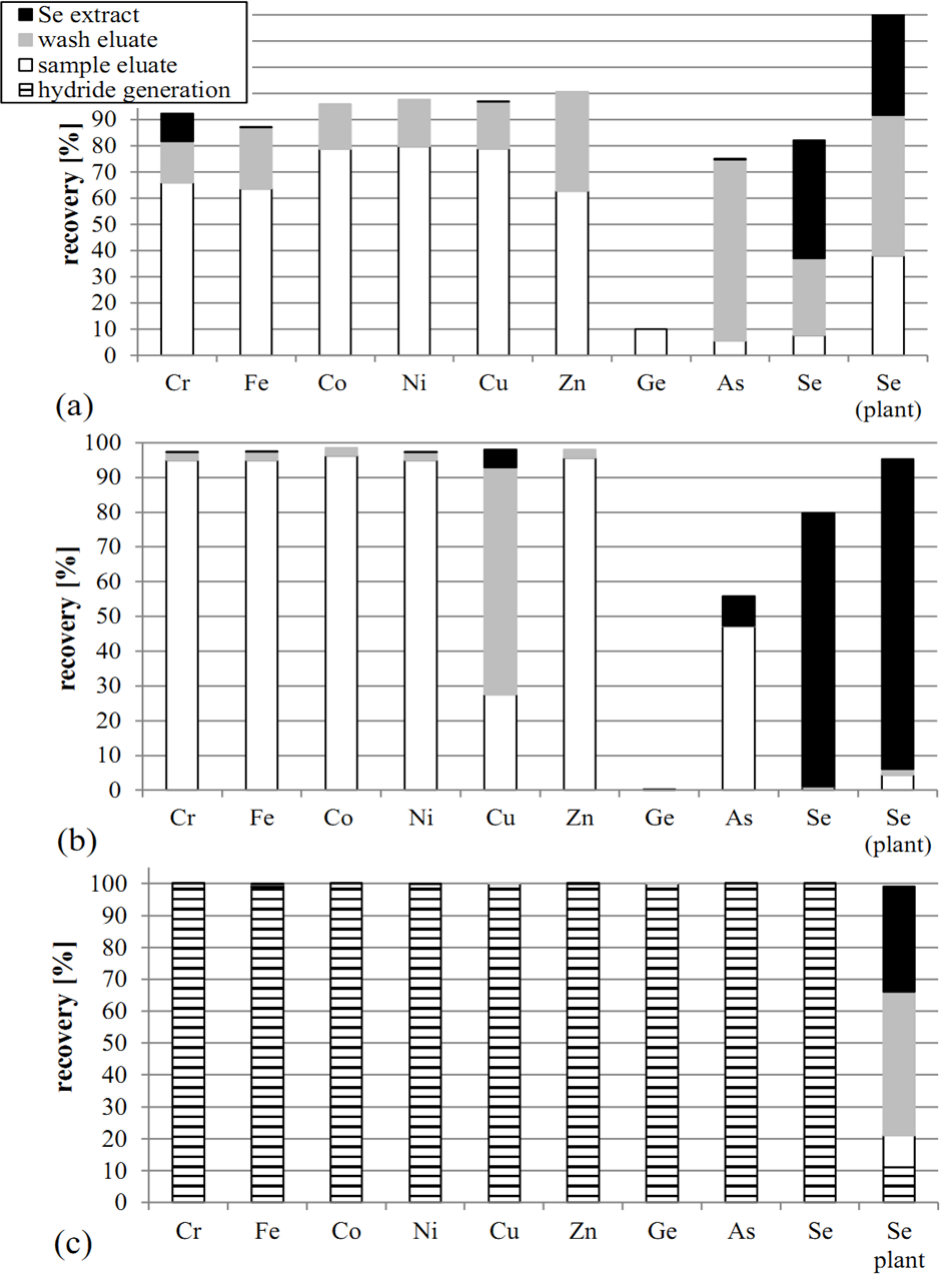
Recoveries (amount quantified in eluates or extracts related to amount added to the column) of critical elements and Se in tests with multi-element solution (column 1–9) differentiated in procedural steps (*sample eluate*, *wash eluate*, *Se extract* and *hydride generation*) and Se recoveries using plant digests with elevated Se concentrations (column 10, “Se plant”). (a) Recoveries in purification method CAE (A). (b) Recoveries in purification method CTR (B). (c) Recoveries in purification method HGT (C).

The identification of individual removal pathways of elements during purification is of special interest in order to evaluate the suitability of the different methods to deal with high amounts of disturbing elements and to detect critical process steps for particular sample matrices. Thereby a more profound statement on the suitability of sample purification methods could be generated for solutions with particular elements to be separated from. To figure out individual removal pathways of elements, the results of the multi-element standard solutions were taken into consideration. Fig 3 illustrates element concentration measured in the *sample eluate*, *wash eluate* and *Se extract* solutions, (a) for CAE, (b) for CTR and (c) for HGT. The latter method additionally includes the phase directly after hydride generation (HG). In this study we focused on the most critical elements Cr, Fe, Co, Ni, Cu, As and Ge as they either inhibit hydride generation or form isobaric interferences. The Se recoveries within the *sample eluate*, *wash eluate* and *Se extract* solutions are additionally illustrated for plant samples.

In CAE (Fig 3(A)), the major load of 63 to 86% of all elements determined—except As, Ge and Se—was recovered in the *sample eluate* indicating that these elements stayed in solution and did not significantly interact with the column packing material. The residual fraction was found in the *wash eluate* and therefore washed out with H_2_O, which indicates that it was not bound to the resin of the column, at most slightly adsorbed or retained in dead end pores or slower transport channels. Except of Cr, only negligibly fractions of the matrix elements were found in the *Se extract* solution assuming that the similar geochemical properties of chromate and selenate–with higher affinity of Se to the resin–caused the relatively high residues. Therefore, AG1-X8 is suitable for Cr separation as well, if Se concentrations are low, as shown by [43]. Arsenic was found by 5% in the *sample eluate* indicating that the most oxidized As species arsenate (H_2_AsO_4_^-^ and HAsO_4_^2-^ at neutral pH) partly adsorbed to the resin due to its double negative charge in analogy to selenate. Therefore AG1-X8 is suitable for arsenate retention as well and was successfully applied by several studies [44, 45]. However, As was removed by almost 70% in the *wash* step. Likely reasons are the removal of H_2_AsO_4_^-^ due to its weaker sorption affinity and the tendency of As to form ferric arsenate complexes with Fe that do not interact with the packing material anymore [46]. This is very likely to play a major role as Fe was removed by 23% in this step. Germanium was only removed by 10% in the *sample eluate*, which indicates that the major fraction was still retained in the column. [47] reported the extensive co-precipitation of Ge with iron oxy(hydr)oxides formed during Fe(II) oxidation or by Fe(III) hydrolysis in neutral solutions that led to the formation of high Ge incorporations into solid Fe phases. CAE purification includes similar conditions and processes, therefore huge amounts of Ge might be retained in precipitates remaining in dead end pores or tiny flow channels within the column. Consequently, CAE might cause problems for samples with high Cr, As and potentially Ge concentrations as all of them are analytically critical elements. Natural plants usually contain all three elements only at trace level unless they grew on highly contaminated sites. In analogy to As, Se tends to co-precipitate with Fe as well [48], which might be a reason for the relatively high Se removal of 30% in the *wash* step. Thus, Fe occurrence plays a major role for the purification efficiency of CAE.

In CTR (Fig 3(B)) all matrix elements added, except Cu, As and Ge, were removed by >96% in the *sample eluate*, and by the remaining few percent in the *wash* step. Copper was retained in the column by >70%, but the major part (65%) was discharged in the *wash eluate*. According to [49] and [50] Cu has a high affinity to form soluble (CuCl_4_)^2-^ complexes. In addition, Cu prefers the formation of thiol-Cu complexes in the presence of 1M HCl whereas other thiol prone metals (e.g. Cr, Fe, Co) already form soluble chloride complexes. Under strong acidic conditions (6M HCl *wash* solution), the formation of (CuCl_4_)^2-^ is advantageous compared to thiol-Cu bonds. Arsenic was removed by less than 50% into the *sample eluate* with no significant amount in the *wash* step and residues of 10% in the *Se extract*. The remaining 40% probably stayed bound to the TCP as it likely applies for over 90% of the Ge. Aside from Se, Ge and As were previously reported to be prone to thiol groups [31]. They form similar bonds and are partly remobilized together with Se or stay bound even after extraction. Intensifying the extraction process e.g. by stronger acids, prolonged boiling time or a third extraction step would probably increase the Se recovery, but also mobilize Ge and As and increase the TOC content by cellulose degradation. In 1M HCl, Fe is available as Fe^2+^ [51] and tends to form chloride complexes rather than binding to thiol groups or precipitation as oxyhydroxide [49]. That is why no significant Fe co-precipitation effects could be observed in contrast to CAE. The Se losses of this purification method, determined in *sample* and *wash eluates*, were slightly higher for plant digests than for standard solutions, which might be caused by non-Se(IV) fractions (Se(VI) or org. Se). However, Se extraction was about 10% higher for plant digests, probably caused by the lower Se, Ge, As and metal concentration and therefore no relevant competition on thiol binding spaces.

In HGT (Fig 3(C)) all matrix elements added were fully removed (i.e., not transferred to the trap) in the *hydride generation* step, including the hydride forming elements As, Ge and Se. [38] performed comprehensive tests with hydride inhibiting elements regarding Se. They found that hydride generation was very sensitive on high concentrations of Fe, Co, Ni and Cu, which suppress Se hydride formation to a minimum (cf. section on Se recoveries). One probable reason for this is the catalytic decomposition of the NaBH_4_, being essential for hydride generation, in the presence of Co(II), Cu(II), Fe(III) and Ni(II) [52]. Additionally, precipitation of those metals and subsequent capture and decomposition of H_2_Se might cause low Se recoveries [38]. The multi-element standard solution contained concentrations of these elements available in their reduced specifications and beyond the tolerable range for Co, Ni and Cu (Table E in S1 File), which very likely contributed to low amounts of Se, As and Ge in the gas trap. Higher acidic conditions (HCl) in the initial sample could reduce this negative effect, because metals will stay in solution to a higher ratio [38]. An additional inhibiting effect might be caused by As(III) concentrations that were elevated by a factor of 10 beyond the tolerance range (Table E in S1 File) reported by [53]. For Se free plant digests doped with Se standard, on average 89% of Se was recovered by hydride generation, being distributed to *sample eluate*, *wash eluate* and Se *extract* during the subsequent anion exchange step. HGT was shown to work efficiently for organic samples, but not for samples with high Fe, Co, Ni, Cu or As contents. For those samples, a previous anion exchange or thiol retention step (in analogy to CAE and CTR) might be helpful as it will probably decrease metals below the critical limits defined by [53] and [38].

In the *Se extract* solutions, the residues of matrix elements are relatively low compared to initial concentrations and laboratory blanks (Table 2 and Table E in S1 File). Only few elements of single sample solutions significantly exceeded the *tolerance test* (Table E in S1 File). This applies for Cr, Fe, Cu, Ge and As in the multi-element standard solution, which does not represent natural samples as it was over-concentrated to figure out removal pathways. Those elements are usually not available in significant amounts in plants and phytoagar. CTR generally provides higher matrix element residues than CAE and HGT due to the boiling extraction, which mobilizes trace elements from the cellulose powder. Slight impurities in the NaOH used for gas trapping likely caused elevated residues of some metals within HGT. If necessary, residues could be further reduced by using suprapure NaOH, e.g. if the sample itself contains high amounts of critical metals. However, any method supplies sufficient purification from matrix elements for Se isotope analytics of natural samples. Nevertheless matrix element residues should be continuously monitored, especially for samples with elevated concentrations in Cr, Fe, Cu, Ge or As.

**Table 2:** Validation tests with Se-free plant digests and phytoagar extracts doped with 300 ng NIST3149 and purified according to CAE, CTR and HGT (with external reproducibility).

	CAE		CTR		HGT*	
sample matrix	δ^82/76^Se [‰]	n	δ^82/76^Se [‰]	n	δ^82/76^Se [‰]	n
**plant** **	n/a		11.6 ± 10.5	6	0.6 ± 0.7	2
**plant** ***	2.0 ± 1.6	2	25.5 ± 5.7	6	0.2 ± 0.2	8
**phytoagar**	5.5 ± 1.2	2	28.4 ± 7.9	2	1.0 ± 0.1	2

*including anion exchange step

**Double Spike added before digestion

***Double Spike added after digestion

### Reliability and validity of developed methodical basis

#### Isobaric interferences formed by sample impurities

A critical error source for Se isotope analytics are very small polyatomic interferences on Se masses formed from the sample carrier solution, the plasma gas (and atmospheric gases that interact with the plasma) and methane. Those molecular disturbances are formed from substances that are essential for the instrumental analytics and not associated with sample properties. Thus they cannot be filtered out in advance, but are accounted for by subtraction of on-peak-zero measurements on Se masses of pure sample carrier solutions (i.e. 2 M HCl) before and after each sample analysis to correct random plasma changes and fluctuations [35]. Other sources of interference are element residues from purification. To quantify their influence, the samples with highest residue concentrations were measured on MC-ICP-MS (*tolerance test*, Table E in S1 File). Signal intensities derived from this measurement were compared to background signals of suprapure 2M HCl blanks measured with MC-ICP-MS. Absolutely no differences could be observed when comparing signal intensities from analytical blanks and samples. This is probably due to efficient filtration of on-line hydride generation, connected to MC-ICP-MS [35]. Because the highest concentrated sample used covers all of the three purification methods tested, all of them can be considered suitable to purify highly concentrated samples from their element residuals in order to avoid isobaric interferences and signal inhibitions.

#### Influence of organic matrix on method validity

The validation test, in which organic matrices were doped with the Se standard solution NIST3149, revealed the high impact of organic residues on method validity. Table 2 shows δ^82/76^Se values determined in these test samples and their external reproducibility with 0 ‰ being the target value for the Se isotopic composition of the internationally accepted δ-zero standard NIST3149. Using CAE, the deviation from 0 ‰ was significant but poorly reproducible with δ^82/76^Se values of 0.42 and 3.58 ‰. This observation applies for phytoagar samples even more than for plant digests (4.32 and 6.74 ‰). CTR treated samples show even higher deviations, whereby the phytoagar values (32.29 and 37.07 ‰) exceed the plant values (-3.22 to 35.98 ‰) again (Table I in S2 File). Plant samples in which the Double Spike was added directly prior to digestion in the microwave beakers had an average δ^82/76^Se value closer to 0 ‰, but with lower external reproducibility than the samples in which the Double Spike was added directly after the samples were cooled down from digestion (Table I in S2 File). However, the validation test clearly failed for both methods and matrices. δ^82/76^Se values determined in samples purified with HGT were very close to 0 ‰ with good external reproducibility for plant samples spiked after digestion. The ones spiked before resulted in δ^82/76^Se values not greatly deviating from 0 ‰, but with weaker reproducibility (Table 2). This is probably due to unpredictable and irregular evaporation and volatilization of Se during digestion. This only concerns a very small amount of Se, but the mentioned processes are highly critical to isotope fractionation and will overproportionally influence the Double Spike correction. Results for phytoagar samples are reproducible, but with a certain deviation from the target value (Table 2). As significant Se contamination from external sources could be excluded, the reason for the high deviations is very likely mass bias by sample preparation and/or instrumental analytics.

**Fig 4:**
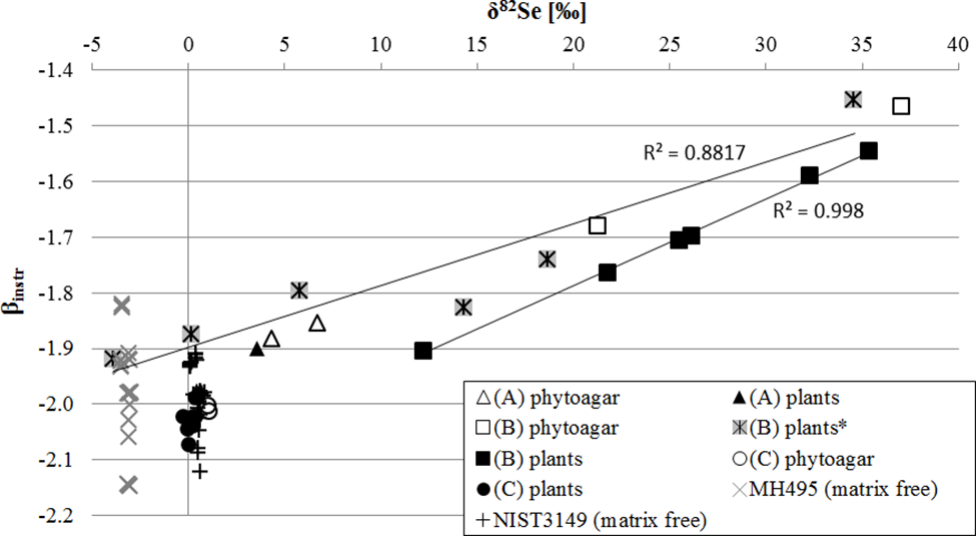
Correlation between Se isotope ratio and β_instr_ of validation tests performed using CAE (A), CTR (B) and HGT (C) as well as NIST3149 and MH495 standard solutions (*Double Spike added prior to digestion, else directly afterwards) (data Table F in S1 File). The Double Spike deconvolution assumes the detected signals are purely due to Se ions and that the shift in the abundance of the Se signals is mass-dependent. The β_instr_ value is thereby calculated by an iterative approach using the 4 Se signals (74, 77, 78, 82).

The instrumental and purification-induced mass bias, which is corrected using the Double Spike, is determined via the β_instr_ factor. During the Double Spike deconvolution this factor is calculated by an iterative approach using the four Se masses ^74^Se, ^77^Se, ^78^Se and ^82^Se. Thereby the β_instr_ varies to a certain extent, which usually has no influence on the δ^82/76^Se value after mass bias correction [36]. However, Fig 4 shows that there is a clear correlation between δ^82/76^Se and β_instr_ in our data, which is most obvious for the plant samples treated with CTR, but also detectable to some extent for CAE treated samples and phytoagar matrices. Although there is a variation in β_instr_ for HGT samples as well, no correlation with δ^82/76^Se could be detected as it applies for matrix free standards (NIST3149, MH495) (Fig 4). This indicates that there is a clear matrix influence with CAE and CTR treated samples, which is not present in samples purified with HGT. This matrix influence is very likely to induce the high deviation of δ^82/76^Se from the “true” value of 0 ‰.

According to [35] the Double Spike method allows correction of instrumental and purification-related mass bias for a wide range of Double Spike/sample proportions, if Double Spike-Se is fully equilibrated with sample-Se. In contrast to HGT and standard solutions, CAE and CTR samples were characterized by significant organic residues, which might have prevented full Double Spike-sample equilibration. Selenium tends to interact and bind to organic matter [39, 54] and is difficult to be equilibrated with the inorganic Double Spike-Se afterwards. Therefore, incomplete equilibration might have impacted the δ^82/76^Se results of samples purified with CAE and CTR.

However, insufficient equilibration might be responsible for some of the shifts in the instrumental mass bias, but cannot nearly explain the observed extent (Fig 4). These large deviations from 0 ‰ are clearly due to polyatomic isobaric interferences on one or several of the four Se masses used for Double Spike deconvolution. As described above, the instrumental mass bias as well as the δ^82/76^Se values of the samples are calculated by an iterative approach using the four Se masses ^74^Se, ^77^Se, ^78^Se and ^82^Se [36]. It presupposes that all signals have been fully corrected for single- and polyatomic mass interferences and that the detected signals are exclusively induced by Se ions. Thus, the Double Spike starts from the premise that the shifts in the abundance of the Se signals are due to mass-dependent instrumental (including purification-related) and mass-dependent natural fractionation processes. Uncorrected isobaric interferences will lead to a completely wrong instrumental mass bias assumed in the instrumental mass bias correction via Double Spike, which in consequence leads to wrong calculated δ^82/76^Se values for the samples. The exceptionally large shifts were only observed in the samples from CAE and CTR, in which residual organic compounds were detected (Tables 1 and 2, Fig 4). [41] and [42] previously described the formation and transportation of organic hydrides in gaseous form to the plasma by cold vapor hydride generation devices. We thus speculate that isobaric interferences on one or several Se masses by such compounds induced the falsification of the instrumental mass bias and of the δ^82/76^Se values. Those are most likely caused by small amounts of organic aerosols produced in the hydride generator and transported into the plasma of the mass spectrometer together with Se hydrides. Additionally, under strongly reducing conditions in the hydride generator, organic aerosols might react with CO_x_ and NO_x_ ubiquitary available in the air. Under changing conditions in the plasma complex polyatomic molecules could be formed from those products, organic aerosols, Se hydrides and other available compounds (Ar, C, H, O), some of which might interfere with Se masses. However, no clear correlation between TOC and δ^82/76^Se or β_instr_ could be found due to the complexity of processes taking place at sample preparation and instrumental analytics with regard to organic matter and Se. Therefore it is almost impossible to clearly identify the composition of such polyatomic organic compounds and their composition is likely to differ from sample to sample. A mathematical correction of these interferences will be very unlikely to work properly. However, with the results of HGT (Fig 4) we were able to show that it is possible to remove organic compounds to an extent that avoids or minimizes the falsification effects and results reliable and valid δ^82/76^Se values. Different from on-line hydride generation, HGT contains a gas phase trapping step in peroxide solution. Organic hydrides and aerosols formed in the hydride generator are likely to remain associated to the gas phase and not to be trapped in H_2_O_2_ and NaOH. They will not be available in the prepared samples anymore when on-line hydride generation and instrumental analytics is performed, and subsequently cannot induce the formation of interferences as described above. Using the HGT method with NIST3149 addition, the “true” value of 0 ‰ in δ^82/76^Se was indeed obtained with an external reproducibility of ±0.2 ‰ (Table 2). This chemical purification therefore constitutes a promising method for accurate and precise Se isotope determinations in natural plants (cf. section on applicability to cultivated plant systems). The precision of 1.0 ±0.1 ‰ for phytoagar extracts should be further improved e.g. by increased organic destruction at an early stage of sample preparation.

The Se isotope determination of the naturally Se containing reference material wheat fluor (NIST1567a) resulted in an average δ^82/76^Se value of +0.27 ±0.08 ‰ (n = 2) with no detectable TOC and very good external reproducibility, which can be used as reference value for internal and external future studies on Se isotope signatures in plants.

### Applicability to and significance for cultivated plant systems

From previously published studies, three data points are available that describe the isotope difference between growth medium and plant. [10] reported a depletion of heavy isotopes of -1.5 ‰ in δ^82/76^Se at plant uptake, whereas [24] found an enrichment of heavy isotopes in plants of +2.5 and +3.2 ‰ in δ^82/76^Se. Regardless of high ecosystem influence and complexity of in situ conditions, these data show that a precision of 0.2 ±0.2 ‰ reached with our developed methods is likely sufficient to resolve Se transformation processes at Se uptake. Spot test results from the minimum parameter plant cultivation approach confirm the sufficiency of precision even for plant internal processes. The Se isotope fractionation Δ^82/76^Se at root-to-shoot translocation according to the transient Rayleigh model was calculated. This includes the δ^82/76^Se measured in roots and shoots after cultivation as well as the Se transfer between these compartments (1-f) (Table 3) (Δ^82/76^Se [‰] = 1000*ln(α); f^(α-1)^ = (δ^82/76^Se_plant,root,shoot_ / δ^82/76^Se_initial_). The largest differences between shoots and roots were obtained from selenate supplied cultivation batches with Δ^82/76^Se values of +3.5 ‰ for 500 μg L^-1^ initial Se and +2.3 ‰ for 1000 μg L^-1^ initial Se in the growth medium respectively. Selenite supplied cultivates were characterized by slightly lower Δ^82/76^Se values of +1.9 ‰ for 500 μg L^-1^ initial Se and +1.2 ‰ for 1000 μg L^-1^ initial Se in the growth medium respectively. Different isotope fractionations among the Se species were very likely caused by plant internal reduction processes and the selective distribution of Se species among the plant parts. Isotopically lighter compounds tend to earlier reduction and incorporation in the roots, whereas heavier compounds remain stable and mobile as selenate and are rather translocated to the shoots. Selenite is generally more prone to reduction than selenate due to different kinetic stability [39]. The fractionation effect decreases with higher Se concentrations supplied because of the increasing role of other transformation mechanisms. However, all determined Se isotope differences are by far higher than the accuracy determined for the method developed in this study for plant samples. The results of the minimum parameter cultivation strongly indicate that the precision is sufficient for this approach, on which basis further experiments can be designed. Furthermore it is applicable to low amounts of Se being transported as it frequently occurs in natural environments.

**Table 3:** Se isotope ratios determined in the system compartments for cultivation batches with selenate supplied in 500 and 1000 μg L^-1^ concentrations and selenite supplied in 500 and 1000 μg L^-1^ concentrations as well as the fraction remaining in the roots after root-shoot translocation f and the Se isotope fractionation during root-shoot transfer Δ^82/76^Se. δ^82/76^Se values are given in relation to NIST3149 (average of measurement before and after sample).

Se species and concentration supplied	System compartment	δ^82/76^Se [‰]*	f (root-shoot)	Δ^82/76^Se (root-shoot) [‰]**
Selenate 500 μg L^-1^	root	-3.19	0.16	3.5
	shoot	-1.95		
Selenate 1000 μg L^-1^	root	-2.85	0.56	2.3
	shoot	-1.11		
Selenite 500 μg L^-1^	root	-1.21	0.61	1.9
	shoot	0.28		
Selenite 1000 μg L^-1^	root	-1.00	0.62	1.2
	shoot	-0.07		

*internal analytical error <0.1 for all samples

**calculated according to the transient Rayleigh model

## Conclusions

In this study a comprehensive method for the preparation and purification of organic rich samples for Se isotope analytics was developed, implemented and validated. The digestion procedure after [30] was proved to be highly suitable for the particular demands of plant material, whereas phytoagar could only be treated appropriately with vacuum filtration. Although all of the three tested purification methods efficiently removed matrix elements and sufficiently recovered Se, HGT was the only procedure yielding valid δ^82/76^Se data. Only this method was able to remove organic residues completely, which turned out to be critical for method validity. It is noteworthy that metal rich samples require another, previous reduction of metal concentrations, e.g. CAE or CTR depending on the element composition. Sufficient accuracy and precision of δ^82/76^Se for plant samples of 0.2 ±0.2 ‰ documents a promising preparation method for the detection of plant internal Se isotope variations, which was shown with selenate and selenite supplied rice plants in each two source concentrations. The accuracy of 1.0 ±0.1 ‰ in the δ^82/76^Se for phytoagar might be further improved with increased organic destruction at a very early stage of sample treatment.

## Supporting information

S1 File Data bases for Fig 1, Fig 2, Fig 3 and Fig 4 including external and internal reproducibility as well as matrix element residuals from CAE, CTR and HGT purification methods. (DOCX)

S2 File Minimal data set underlying the results described in this manuscript. (DOCX)
