# Supplementary material for: Preparation and purification of organic samples for selenium isotope studies
Source: PLoS One. 2018 Mar 6;13(3):e0193826. doi: 10.1371/journal.pone.0193826 (PMC5839574; doi:10.1371/journal.pone.0193826)
Supplement: S1 File — (DOCX) [file pone.0193826.s001.docx]

# Banning et al. PLOS ONE

# Supporting Information S1 – Data bases for Fig 1, Fig 2, Fig 3 and Fig 4 including external and internal reproducibility as well as matrix element residuals from CAE, CTR and HGT purification methods

**Table A. Data basis of Fig 1 – digestion (modification after Kopp, 1999) and vacuum filtration of phytoagar (reference: NIST1567a recovery in digestion: 98.0 %)**

| **Se added** | **selenate** | | | **selenite** | | | **SeMet** | | | **average** |
| --- | --- | --- | --- | --- | --- | --- | --- | --- | --- | --- |
| **[µg L^-1^]** | **100** | **500** | **1000** | **100** | **500** | **1000** | **100** | **500** | **1000** |  |
| **digestion**  **Se recovery [%]** | 77.5  ± 9.2 | 84.8  ± 7.6 | 81.2  ± 4.0 | 58.3  ± 1.5 | 76.0  ± 3.0 | 72.0  ± 4.1 | 76.5  ± 0.6 | 70.3  ± 4.5 | 71.8  ± 4.9 | 74.3  ± 5.5 |
| **n** | 2 | 2 | 2 | 2 | 2 | 2 | 2 | 2 | 2 | 18 |
| **vacuum filtration**  **Se recovery [%]** | 102.5  ± 0.6 | 105.3  ± 0.6 | 107.6  ± 0.2 | 102.6  ± 0.5 | 108.8  ± 0.3 | 102.5  ± 0.9 | 101.2  ± 2.5 | 101.7  ± 3.6 | 97.7  ± 1.9 | 103.3  ± 2.6 |
| **n** | 3 | 3 | 3 | 3 | 3 | 3 | 5 | 5 | 5 | 33 |

**Table B. Data basis of Fig 2 (a) – matrix removal pathways of CAE purification method (reference: CRM-TMDW recovery ranges between 100 and 105 % depending on the element, 103 % for Se)**

| **Matrix element** | **sample eluate solution [%]** | **wash eluate solution [%]** | **Se extract solution [%]** | **total removed from column [%]** | **deficit [%]** |
| --- | --- | --- | --- | --- | --- |
| **Cr** | 65.91 ± 1.92 | 15.66 ± 1.13 | 10.64 ± 1.59 | 92.21 | 7.79 |
| **Fe** | 63.64 ± 4.41 | 23.25 ± 2.21 | 0.22 ± 0.48 | 87.11 | 12.89 |
| **Co** | 78.59 ± 1.74 | 17.28 ± 1.60 | LDL | 95.87 | 4.13 |
| **Ni** | 79.71 ± 1.77 | 17.81 ± 1.66 | LDL | 97.52 | 2.48 |
| **Cu** | 78.81 ± 1.67 | 17.94 ± 1.65 | 0.02 ± 0.01 | 96.77 | 3.23 |
| **Zn** | 62.66 ± 4.63 | 37.94 ± 5.1 | LDL | 100.6 | - |
| **Ge** | 9.92 ± 6.65 | LDL | LDL | 9.92 | 90.08 |
| **As** | 5.46 ± 1.64 | 69.09 ± 8.56 | 0.45 ± 0.28 | 75 | 25 |
| **Se** | 7.55 ± 3.00 | 29.36 ± 5.14 | 45.14 ± 6.98 | 82.05 | 17.95 |
| **Se plant** | 38.1 ± 38.8 | 53.6 ± 48.9 | 40.4 ± 19.6 | 132.1 | - |

**Table C. Data basis of Fig 2 (b) – matrix removal pathways of CTR purification method (reference: CRM-TMDW recovery ranges between 98 and 103 % depending on the element, 99 % for Se)**

| **Matrix element** | **sample eluate solution [%]** | **wash eluate solution [%]** | **Se extract solution [%]** | **total removed from column [%]** | | **deficit [%]** |
| --- | --- | --- | --- | --- | --- | --- |
| **Cr** | 94.89 ± 2.32 | 2.31 ± 0.35 | 0.01 ± 0.01 | 97.21 | 2.79 | |
| **Fe** | 95.01 ± 2.45 | 2.35 ± 0.33 | 0.05 ± 0.01 | 97.41 | 2.59 | |
| **Co** | 96.22 ± 2.24 | 2.33 ± 0.35 | LDL | 98.55 | 1.45 | |
| **Ni** | 94.92 ± 1.92 | 2.34 ± 0.38 | 0.01 ± 0.00 | 97.27 | 2.73 | |
| **Cu** | 27.56 ± 15.6 | 65.31 ± 12.87 | 5.11 ± 3.40 | 97.98 | 2.02 | |
| **Zn** | 95.59 ± 2.03 | 2.46 ± 0.34 | LDL | 98.05 | 1.95 | |
| **Ge** | 0.01 ± 0.01 | 0 ± 0 | 0.22 ± 0.14 | 0.23 | 99.77 | |
| **As** | 47.18 ± 15.34 | 0.09 ± 0.06 | 8.65 ± 4.31 | 55.92 | 44.08 | |
| **Se** | 0.81 ± 0.52 | 0.2 ± 0.07 | 78.78 ± 13.91 | 79.79 | 20.21 | |
| **Se plant** | 4.5 ± 2.5 | 1.6 ± 1.4 | 89.1 ± 19.3 | 95.2 | 4.8 | |

**Table D. Data basis of Fig 2 (c) – matrix removal pathways of HGT purification method (reference: CRM-TMDW recovery ranges between 94 and 110 % depending on the element, 105 % for Se)**

| **Matrix element** | **residual from HG [%]** | **sample eluate solution [%]** | **wash eluate solution [%]** | **Se extract solution [%]** | **total removed from column [%]** | **deficit [%]** |
| --- | --- | --- | --- | --- | --- | --- |
| **Cr** | 99.99 ± 0.00 | 0.00 ± 0.00 | 0.00 ± 0.00 | 0.04 ± 0.00 | 100.02 | - |
| **Fe** | 99.14 ± 0.01 | 0.50 ± 0.03 | 0.10 ± 0.01 | 0.01 ± 0.00 | 99.74 | 0.26 |
| **Co** | 100.00 ± 0.00 | 0.00 ± 0.00 | 0.00 ± 0.00 | 0.00 ± 0.00 | 100.00 | 0.00 |
| **Ni** | 99.86 ± 0.00 | 0.00 ± 0.00 | 0.00 ± 0.00 | 0.06 ± 0.00 | 99.92 | 0.08 |
| **Cu** | 100.00 ± 0.00 | 0.00 ± 0.00 | 0.00 ± 0.00 | 0.00 ± 0.00 | 100.00 | 0.00 |
| **Zn** | 99.75 ± 0.03 | 0.20 ± 0.02 | 0.03 ± 0.00 | 0.01 ± 0.00 | 99.98 | 0.02 |
| **Ge** | 99.99 ± 0.00 | 0.00 ± 0.00 | 0.00 ± 0.01 | 0.00 ± 0.00 | 99.99 | 0.01 |
| **As** | 99.96 ± 0.00 | 0.00 ± 0.00 | 0.01 ± 0.00 | 0.03 ± 0.00 | 100.00 | 0.00 |
| **Se** | 99.92 ± 0.00 | 0.00 ± 0.00 | 0.03 ± 0.01 | 0.05 ± 0.01 | 100.01 | - |
| **Se plant** | 11.00 ± 0.10 | 10.09 ± 0.50 | 45.19 ± 1.20 | 33.02 ± 0.84 | 99.30 | 0.70 |

**Table E. Matrix residuals in purified samples (Se extracts) processed with CAE, CTR and HGT for various matrices – MS multi-element solution, ip Indian plants, p phytoagar, cp cultivated plants (sample volumes 10 mL each) (reference: CRW-TMDW recoveries see Tables B and D)**

| **[ng]** | **n** | **Cr** | **Fe** | **Co** | **Ni** | **Cu** | **Zn** | **Ge** | **As** |
| --- | --- | --- | --- | --- | --- | --- | --- | --- | --- |
| **CAE_MS_** | 9 | 10640 ± 1590 | 223 ± 483 | <0.02 | 4.8 ± 1.7 | 18.5 ± 14.7 | 12.4 ± 3.9 | <0.1 | 44.9 ± 27.8 |
| **CAE_pp_** | 9 | 1.9 ± 2.3 | 30.7 ± 18.4 | <0.02 | 1.7 ± 1.1 | 2.6 ± 1.8 | 58.3 ± 41.3 | <0.1 | <0.1 |
| **CAE_p_** | 18 | 1.1 ± 0.8 | 29.2 ± 26.5 | <0.02 | 0.1 ± 0.9 | 0.9 ± 2.2 | 10.5 ± 18.3 | <0.1 | 0.6 ± 0.5 |
| **CAE_cp_** | 9 | 0.5 ± 0.5 | 45.8 ± 35.2 | <0.2 | 0.0 ± 0.1 | 3.1 ± 2.8 | 124 ± 180 | <0.1 | 0.4 ± 0.4 |
| **CTR_MS_** | 9 | 7.8 ± 7.2 | 47.9 ± 26.4 | 1.31 ± 0.50 | 5.7 ± 3.6 | 5280 ± 326 | 81.7 ± 43.6 | 79.2 ± 73.9 | 2750 ± 2360 |
| **CTR_pp_** | 9 | 0.8 ± 2.3 | 4.1 ± 40.8 | <0.02 | 1.1 ± 0.9 | 0.5 ± 1.1 | 248 ± 248 | <0.1 | 0.7 ± 0.8 |
| **CTR_p_** | 18 | 2.5 ± 2.9 | 137* ± 227 | <0.02 | 1.6 ± 1.0 | 24.5 ± 36.9 | 34.5 ± 193 | <0.1 | 5.4 ± 1.1 |
| **CTR_cp_** | 9 | 0.3 ± 1.3 | 11.7 ± 12.7 | <0.02 | 0.2 ± 0.6 | 1.2 ± 1.0 | 37.8 ± 57.7 | <0.1 | 4.3 ± 1.0 |
| **HGT_MS_** | 2 | 34.7 ± 1.2 | 6.13 ± 1.41 | 0.41 ± 0.20 | 59.0 ± 1.8 | <0.1 | 6.2 ± 0.7 | <0.1 | 2.8 ± 0.0 |
| **HGT_p_** | 2 | 53.8 ± 10.0 | 15.5 ± 5.32 | 0.53 ± 0.00 | 112 ± 57.8 | 0.5 ± 0.3 | 11.1 ± 0.2 | <0.1 | <0.1 |
| **HGT_cp_** | 2 | 35.8 ± 1.6 | 32.1 ± 11.8 | 0.71 ± 0.31 | 174 ± 48.3 | 13.2 ± 8.4 | 9.0 ± 1.7 | 0.4 ± 0.0 | <0.1 |
|  |  |  |  |  |  |  |  |  |  |
| **tolerance test** | **1** | **1.39** | **52.3** | **0.57** | **3.1** | **13.3** | **686** | **0.1** | **4.9** |

*Fe exceeding caused by incidentally high blank, phytoagar extract only contained small traces, for HGT the NaOH blank of Cr, Al, P and Ni was exceptionally high due to p.a. quality (could be improved by using pure chemicals)

**Table F. Data basis of Fig 3 – Se isotope ratios of validation test samples purified with CAE, CTR and HGT, mass bias (β_instr_) and Se recovery**

| **Purification method** | **Sample ID** | **matrix** | **DS addition** | **internal error**  (2 SE) | **δ^82^Se**  **[‰]****** | **β_instr_** |
| --- | --- | --- | --- | --- | --- | --- |
| **CAE** | Pl1 A | Se doped plant | ** | 0.03 | 0.42 | -1.98 |
|  | Pl2 A | Se doped plant | ** | 0.06 | 3.58 | -1.90 |
|  | Ag1 A | Se doped phytoagar | ** | 0.02 | 6.74 | -1.85 |
|  | Ag2 A | Se doped phytoagar | ** | 0.02 | 4.32 | -1.88 |
| **CTR** | Pl1 B | Se doped plant | * | 0.07 | 26.11 | -1.71 |
|  | Pl2 B | Se doped plant | * | 0.12 | 12.82 | -1.90 |
|  | Pl3 B | Se doped plant | * | 0.12 | 22.48 | -1.76 |
|  | Pl4 B | Se doped plant | * | 0.09 | 32.94 | -1.59 |
|  | Pl5 B | Se doped plant | * | 0.20 | 35.98 | -1.55 |
|  | Pl6 B | Se doped plant | * | 0.82 | 26.80 | -1.70 |
|  | Pl7 B | Se doped plant | ** | 0.16 | 0.86 | -1.88 |
|  | Pl8 B | Se doped plant | ** | 0.19 | -3.22 | -1.92 |
|  | Pl9 B | Se doped plant | ** | 0.05 | 6.40 | -1.80 |
|  | Pl10 B | Se doped plant | ** | 0.21 | 35.22 | -1.45 |
|  | Pl11 B | Se doped plant | ** | 0.04 | 19.31 | -1.74 |
|  | Pl12 B | Se doped plant | ** | 0.20 | 14.98 | -1.83 |
|  | Ag1 B | Se doped phytoagar | ** | 0.20 | 21.29 | -1.68 |
|  | Ag2 B | Se doped phytoagar | ** | 0.32 | 37.07 | -1.46 |
| **HGT** | Pl1 C | Se doped plant | * | 0.16 | 0.74 | -2.01 |
|  | Pl2 C | Se doped plant | * | 0.34 | 2.10 | -1.95 |
|  | Pl3 C | Se doped plant | ** | 0.03 | 0.00 | -2.04 |
|  | Pl3 C | Se doped plant | ** | 0.02 | 0.00 | -2.05 |
|  | Pl4 C | Se doped plant | ** | 0.02 | 0.02 | -2.07 |
|  | Pl5 C | Se doped plant | ** | 0.05 | 0.40 | -1.99 |
|  | Pl6 C | Se doped plant | ** | 0.05 | -0.24 | -2.02 |
|  | Pl7 C | Se doped plant | ** | 0.04 | 0.37 | -2.02 |
|  | Pl8 C | Se doped plant | ** | 0.03 | 0.34 | -2.02 |
|  | Pl9 C | Se doped plant | ** | 0.04 | 0.34 | -2.04 |
|  | Pl10 C | Se doped plant | ** | 0.05 | 0.33 | -2.04 |
|  | Ag1 C | Se doped phytoagar | ** | 0.04 | 1.04 | -2.01 |
|  | Ag1 C | Se doped phytoagar | ** | 0.05 | 1.04 | -2.00 |
|  | Ag2 C | Se doped phytoagar | ** | 0.07 | 1.13 | -2.01 |
|  | WF1 | NISTSRM1567a | ** | 0.07 | 0.34 | -1.99 |
|  | WF2 | NISTSRM1567a | ** | 0.05 | 0.19 | -2.00 |

*Double Spike added before digestion

**Double Spike added after digestion

****related to the average δ^82/76^Se of NISTSRM3149 measured before and after the respective sample
