# Supplementary material for: Preparation and purification of organic samples for selenium isotope studies
Source: PLoS One. 2018 Mar 6;13(3):e0193826. doi: 10.1371/journal.pone.0193826 (PMC5839574; doi:10.1371/journal.pone.0193826)
Supplement: S2 File — (DOCX) [file pone.0193826.s002.docx]

# Banning et al. PLOS ONE

# Supporting Information S2 – Minimal data set underlying the results described in this manuscript

*List of abbreviations in sample IDs*

| pini | phytoagar initial | |  | |  |
| --- | --- | --- | --- | --- | --- |
| pac | phytoagar after cultivation | | | |  |
| cp | cultivated plants | |  | |  |
| cpr | cultivated plants - roots | |  | |  |
| cps | cultivated plants - shoots | |  | |  |
| I to V | MinPaX |  |  | |  |
| 1 to 10 | Repetition or box | 1 | selenate 100 µg L^-1^ | | |
|  |  | 2 | selenate 500 µg L^-1^ | | |
|  |  | 3 | selenate 1000 µg L^-1^ | | |
|  |  | 4 | selenite 100 µg L^-1^ | | |
|  |  | 5 | selenite 500 µg L^-1^ | | |
|  |  | 6 | selenite 1000 µg L^-1^ | | |
|  |  | 7 | SeMet 100 µg L^-1^ | | |
|  |  | 8 | SeMet 500 µg L^-1^ | | |
|  |  | 9 | SeMet 1000 µg L^-1^ | | |
|  |  | 10 | no Se supplied | | |
| WF | Wheat Flour NISTSRM1567a reference | | | |  |
| MS | multielement standard | | | | |
| PP | Punjab plants | | | | |
| VT | validation test (Se free matrix + NIST-Se) | | | | |
| cp | cultivated plants | | | | |
| p | phytoagar | | |  |  |
| c | clean and condition | | |  |  |
| e | eluate | | |  |  |
| w | wash | | |  |  |
| Se | Se extract | | |  |  |
| A, B, C | purification method GLS (A), CTR (B), HGT (C) | | | |  |
| S | supernatent (after B - centrifugation) | | | |  |
| MinPaX | Minimum Parameter Experiment (plants) | | | |  |
|  |  | | | |  |
|  |  | | |  |  |
|  |  | | |  |  |
|  |  | | |  |  |
|  |  | | | |  |
|  |  | | | |  |

**Table A:** Phytoagar treatment – Se concentration using digestion after Kopp (1999) and vacuum filtration

| ***digestion after Kopp (1999)*** | | **Se in digest [µg L^-1^]** | | | | | | | |
| --- | --- | --- | --- | --- | --- | --- | --- | --- | --- |
| **Se species** | **Se added to phytoagar [µg L^-1^]** | **I** | **II** |  |  |  | | **average** | |
| **selenate** | 100 | 96.0 | 59.1 |  |  |  | | 77.5 ±18.5 | |
|  | 500 | 499 | 348 |  |  |  | | 424 ±75.6 | |
|  | 1000 | 891 | 732 |  |  |  | | 812 ±79.7 | |
| **selenite** | 100 | 55.4 | 61.3 |  |  |  | | 58.4 ±3.0 | |
|  | 500 | 410 | 350 |  |  |  | | 380 ±29.7 | |
|  | 1000 | 801 | 639 |  |  |  | | 720 ±81.2 | |
| **SeMet** | 100 | 75.3 | 77.7 |  |  |  | | 76.5 ±1.2 | |
|  | 500 | 396 | 306 |  |  |  | | 351 ±45.1 | |
|  | 1000 | 815 | 620 |  |  |  | | 718 ±97.5 | |
|  |  |  | | | | | | | |
| ***vacuum filtration*** | | **Se in extract [µg L^-1^]** | | | | | | | |
| **Se species** | **Se added to phytoagar [µg L^-1^]** | **I** | **II** | **III** | **IV** | | **V** | | **average** |
| **selenate** | 100 | 102 | 103 | 103 | n/a | | n/a | | 103 ±0.7 |
|  | 500 | 522 | 530 | 528 | n/a | | n/a | | 526 ±3.0 |
|  | 1000 | 1070 | 1080 | 1080 | n/a | | n/a | | 1080 ±1.7 |
| **selenite** | 100 | 102 | 103 | 107 | n/a | | n/a | | 103 ±0.5 |
|  | 500 | 544 | 542 | 547 | n/a | | n/a | | 544 ±1.7 |
|  | 1000 | 1010 | 1020 | 1040 | n/a | | n/a | | 1030 ±9.3 |
| **SeMet** | 100 | 96.4 | 96.1 | 97.4 | 110 | | 107 | | 101 ±5.5 |
|  | 500 | 478 | 482 | 485 | 573 | | 525 | | 508 ±32.3 |
|  | 1000 | 915 | 901 | 896 | 1140 | | 1030 | | 977 ±88.3 |

**Table B:** Initial Se in NISTSRM1567a measured with EDX and Se concentration after using digestion after Bell et al. (1992) compared to Kopp (1999)

|  | **EDX** | **digestion after Bell et al. (1992)** | | **digestion after Kopp (1999)** | |
| --- | --- | --- | --- | --- | --- |
| **sample No.** | **Se in solid [ppm]** | **Se in digest [µg L^-1^]** | **Se in solid [ppm]** | **Se in digest [µg L^-1^]** | **Se in solid [ppm]** |
| 1 | 1.36 | 9.79 | 0.98 | 9.65 | 0.97 |
| 2 | 1.29 | 6.12 | 0.61 | 9.83 | 0.98 |
| 3 | 1.39 | 7.53 | 0.75 | 9.99 | 1.00 |
| 4 | 0.98 | 7.78 | 0.78 | 8.54 | 0.85 |
| 5 | 1.21 | 7.86 | 0.79 | 9.67 | 0.97 |
| **6** |  | 8.48 | 0.85 | 9.59 | 0.96 |
| 7 |  | 9.28 | 0.93 | 10.0 | 1.00 |
| 8 |  | 9.35 | 0.93 | 7.50 | 0.75 |
| 9 |  | 8.87 | 0.89 | 11.0 | 1.10 |
| 10 |  | 9.02 | 0.90 | 11.2 | 1.12 |
| 11 |  | 8.04 | 0.80 | 11.4 | 1.14 |
| 12 |  | 9.02 | 0.90 | 11.4 | 1.14 |
| 13 |  | 8.44 | 0.84 | 10.9 | 1.09 |
| 14 |  | 8.60 | 0.86 | 10.9 | 1.09 |
| 15 |  | 9.58 | 0.96 |  |  |
| 16 |  | 9.75 | 0.98 |  |  |
| 17 |  | 9.78 | 0.98 |  |  |
| 18 |  | 13.6 | 1.36 |  |  |
| 19 |  | 9.36 | 0.94 |  |  |
| 20 |  | 9.82 | 0.98 |  |  |
| 21 |  | 10.8 | 1.08 |  |  |
| 22 |  | 8.64 | 0.86 |  |  |
| 23 |  | 10.5 | 1.05 |  |  |
| 24 |  | 8.98 | 0.90 |  |  |
| 25 |  | 10.6 | 1.06 |  |  |
| 26 |  | 10.7 | 1.07 |  |  |
| 27 |  | 12.4 | 1.24 |  |  |
| 28 |  | 10.3 | 1.03 |  |  |
| 29 |  | 8.00 | 0.80 |  |  |
| 30 |  | 9.14 | 0.91 |  |  |
| 31 |  | 9.78 | 0.98 |  |  |
| **average** | **1.25 ±0.15** | **9.35 ±1.02** | **0.94 ±0.06** | **10.1 ±0.81** | **1.01 ±0.08** |

**Table C:** Initial organic C content in NISTSRM1567a measured with CSA and TOC residuals in treated phytoagar and plant samples

| **CSA** | | | | | |  | | |  | |  | | |  | | |  |
| --- | --- | --- | --- | --- | --- | --- | --- | --- | --- | --- | --- | --- | --- | --- | --- | --- | --- |
| **Sample** | | | | **C [ppm]** | |  | | |  | |  | | |  | | |  |
| **NISTSRM1567a – 1** | | | | 43.3 | |  | | |  | |  | | |  | | |  |
| **NISTSRM1567a – 2** | | | | 41.4 | |  | | |  | |  | | |  | | |  |
| **NISTSRM1567a – 3** | | | | 43.9 | |  | | |  | |  | | |  | | |  |
| **NISTSRM1567a – 4** | | | | 42.6 | |  | | |  | |  | | |  | | |  |
| **NISTSRM1567a – 5** | | | | 42.6 | |  | | |  | |  | | |  | | |  |
| **average** | | | | 42.8 ±0.69 | |  | | |  | |  | | |  | | |  |
|  | | | | | | | | | | | | | | | | | |
| ***digestion after Kopp (1999)***  (WF – Wheat Flour NISTSRM1567a) | | | | | | | | | | | | | | | | | |
|  | **weight [mg]** | | **TOC**  **[mg L^-1^]** | | **average**  **[mg L^-1^]** | | | **average (blank substracted) [mg L^-1^]** | | | | **C_absolute_**  **[mg]** | | | **C_initial_**  **[mg]** | | **C_residual_**  **[%]** |
| **Blank** | 0 | | 5.5 | | 5.5 ±0.0 | | | - | | | |  | | |  | |  |
|  |  | | 5.5 | |  | | |  | | | |  | | |  | |  |
|  |  | | 5.4 | |  | | |  | | | |  | | |  | |  |
| **WF1** | 106 | | 8.0 | | 8.0 ±0.0 | | | 2.5 ±0.0 | | | | 0.03 | | | 45.5 | | 0.06 |
|  |  | | 8.0 | |  | | |  | | | |  | | |  | |  |
|  |  | | 8.0 | |  | | |  | | | |  | | |  | |  |
| **WF2** | 108 | | 77.4 | | 76.3 ±0.7 | | | 70.8 ±0.7 | | | | 0.71 | | | 46.1 | | 1.54 |
|  |  | | 75.8 | |  | | |  | | | |  | | |  | |  |
|  |  | | 75.7 | |  | | |  | | | |  | | |  | |  |
| **WF3** | 102 | | 64.1 | | 62.0 ±1.4 | | | 56.5 ±1.4 | | | | 0.57 | | | 43.5 | | 1.30 |
|  |  | | 61.5 | |  | | |  | | | |  | | |  | |  |
|  |  | | 60.4 | |  | | |  | | | |  | | |  | |  |
| **WF4** | 103 | | 51.2 | | 51.2 ±0.0 | | | 45.7 ±0.0 | | | | 0.46 | | | 44.2 | | 1.03 |
|  |  | | 51.1 | |  | | |  | | | |  | | |  | |  |
|  |  | | 51.2 | |  | | |  | | | |  | | |  | |  |
| **WF5** | 104 | | 20.1 | | 20.1 ±0.0 | | | 14.6 ±0.0 | | | | 0.15 | | | 44.6 | | 0.33 |
|  |  | | 20.1 | |  | | |  | | | |  | | |  | |  |
|  |  | | 20.1 | |  | | |  | | | |  | | |  | |  |
| **WF6** | 104 | | 25.2 | | 25.1 ±0.1 | | | 19.6 ±0.1 | | | | 0.20 | | | 44.3 | | 0.44 |
|  |  | | 25.1 | |  | | |  | | | |  | | |  | |  |
|  |  | | 24.9 | |  | | |  | | | |  | | |  | |  |
| **WF7** | 102 | | 39.5 | | 39.6 ±0.1 | | | 34.1 ±0.1 | | | | 0.34 | | | 43.4 | | 0.78 |
|  |  | | 39.7 | |  | | |  | | | |  | | |  | |  |
|  |  | | 39.5 | |  | | |  | | | |  | | |  | |  |
| **WF8** | 105 | | 31.1 | | 30.8 ±0.2 | | | 25.3 ±0.2 | | | | 0.25 | | | 44.8 | | 0.57 |
|  |  | | 30.5 | |  | | |  | | | |  | | |  | |  |
|  |  | | 30.7 | |  | | |  | | | |  | | |  | |  |
| **WF9** | 100 | | 10.9 | | 10.9 ±0.0 | | | 5.4 ±0.0 | | | | 0.05 | | | 42.9 | | 0.13 |
|  |  | | 10.8 | |  | | |  | | | |  | | |  | |  |
|  |  | | 10.9 | |  | | |  | | | |  | | |  | |  |
| **average** | | | | | **36.0 ±18.9** | | | **30.5 ±18.9** | | | | **0.31 ±0.19** | | | **44.4 ±7.9** | | **0.69 ±0.42** |
|  |  | |  | |  | | |  | | | |  | | |  | |  |
|  |  | |  | |  | | |  | | | |  | | |  | |  |
|  |  | |  | |  | | |  | | | |  | | |  | |  |
|  |  | |  | |  | | |  | | | |  | | |  | |  |
|  |  | |  | |  | | |  | | | |  | | |  | |  |
|  |  | |  | |  | | |  | | | |  | | |  | |  |
|  |  | |  | |  | | |  | | | |  | | |  | |  |
|  |  | |  | |  | | |  | | | |  | | |  | |  |
|  | | | | | | | | | | | | | | | | | |
|  | | | | | | | | | | | | | | | | | |
| **(continuation of Table C)** | | | | | | | | | | | | | | | | | |
| ***NISTSRM1567a digestion after Bell et al. (1992)* (WF – Wheat Flour NISTSRM1567a)** | | | | | | | | | | | | | | | | | |
|  | | **sample weight [mg]** | | **TOC**  **[mg L^-1^]** | | | **TOC (blank substracted)**  **[mg L^-1^]** | | | **C_initial_ [wt.%]** | | | **C_initial_**  **[mg]** | | | **C_residual_**  **[%]** | |
| **Blank** | | 0 | | 8.8 | | | - | | |  | | |  | | |  | |
| **WF1** | | 100 | | 313 | | | 304 | | | 42.8 | | | 42.8 | | | 7.11 | |
| **WF2** | | 100 | | 300 | | | 291 | | | 42.8 | | | 42.8 | | | 6.80 | |
| **WF3** | | 100 | | 351 | | | 343 | | | 42.8 | | | 42.8 | | | 8.01 | |
| **WF4** | | 100 | | 410 | | | 402 | | | 42.8 | | | 42.8 | | | 9.39 | |
| **average** | |  | | **344 ±37.3** | | | **334 ±37.3** | | |  | | |  | | | **7.83 ±0.87** | |
|  | |  | |  | | |  | | |  | | |  | | |  | |
| ***Cultivated plant digestion after Kopp et al. (1999)* (CP – cultivated plants from MinPaX**  **(complete minimization with Tissue Lyzer))** | | | | | | | | | | | | | | | | | |
|  | | **sample weight [mg]** | | **TOC**  **[mg L^-1^]** | | | **TOC (blank substracted) [mg L^-1^]** | | | **C_initial_ [wt.%]** | | | **C_initial_**  **[mg]** | | | **C_residual_**  **[%]** | |
| **Blank** | | 0 | | 8.8 | | | - | | |  | | |  | | |  | |
| **CP1** | | 1200 | | 5.2 | | | <blank | | | 0.21 | | | 2.48 | | | -1.42 | |
| **CP2** | | 1200 | | 9.0 | | | <blank | | | 0.21 | | | 2.48 | | | 0.08 | |
| **CP3** | | 1220 | | 8.1 | | | <blank | | | 0.21 | | | 2.53 | | | -0.25 | |
| **CP4** | | 1110 | | 7.6 | | | <blank | | | 0.21 | | | 2.30 | | | -0.52 | |
| **average** | |  | | **7.5 ±1.1** | | | **<blank** | | |  | | |  | | | **<0.04** | |
|  | |  | |  | | |  | | |  | | |  | | |  | |
| ***Cultivated plant digestion after Kopp et al. (1999)* (CP – cultivated plants from MinPaX**  **(incomplete minimization))** | | | | | | | | | | | | | | | | | |
|  | | **sample weight [mg]** | | **TOC**  **[mg L^-1^]** | | | **TOC (blank substracted)**  **[mg L^-1^]** | | | **C_initial_ [wt.%]** | | | **C_initial_**  **[mg]** | | | **C_residual_**  **[%]** | |
| **Blank** | | 0 | | 8.8 | | | - | | |  | | |  | | |  | |
| **CPI1** | | 100 | | 88.9 | | | 80.1 | | | 43.8 | | | 43.8 | | | 1.83 | |
| **CPI2** | | 70 | | 205 | | | 196 | | | 43.8 | | | 30.6 | | | 6.40 | |
| **CPI3** | | 100 | | 238 | | | 229 | | | 43.8 | | | 43.8 | | | 5.23 | |
| **CPI4** | | 100 | | 216 | | | 208 | | | 43.8 | | | 43.8 | | | 4.74 | |
| **average** | |  | |  | | | **178 ±49.0** | | |  | | |  | | | **4.55 ±1.36** | |
|  | |  | |  | | |  | | |  | | |  | | |  | |
|  | | | | | | | | | | | | | | | | | |
| **Residual TOC in phytoagar (P) extracts (vacuum filtration)** | | | | | | | | | | | | | | | | | |
|  | |  | | **TOC**  **[mg L^-1^]** | | | **TOC (blank substracted)**  **[mg L^-1^]** | | |  | | |  | | |  | |
| **P1** | |  | | 253 | | | 262 | | |  | | |  | | |  | |
| **P2** | |  | | 394 | | | 403 | | |  | | |  | | |  | |
| **P3** | |  | | 304 | | | 313 | | |  | | |  | | |  | |
| **average** | |  | |  | | | **326 ±51.5** | | |  | | |  | | |  | |

**Table D:** Matrix element and Se concentrations measured in multi-element standard and plants from Punjab for each step of method (A) purification and ubiquitary anions present in samples before addition to column

| ***initial plant digests (applies for (B) as well)*** | | | | | | | | | | | | | | | | |
| --- | --- | --- | --- | --- | --- | --- | --- | --- | --- | --- | --- | --- | --- | --- | --- | --- |
|  | **Amount taken** | **Na** | **Mg** | **Al** | **P** | **Ca** | **Cr** | **Fe** | **Co** | **Ni** | **Cu** | **Zn** | **Ge** | **As** | **Se** | |
| **Digest** | **[µL]** | **[µg L^-1^]** | **[µg L^-1^]** | **[µg L^-1^]** | **[µg L^-1^]** | **[µg L^-1^]** | **[µg L^-1^]** | **[µg L^-1^]** | **[µg L^-1^]** | **[µg L^-1^]** | **[µg L^-1^]** | **[µg L^-1^]** | **[µg L^-1^]** | **[µg L^-1^]** | **[µg L^-1^]** | |
| **1** | 675 | 16600 | 14600 | 2000 | 12600 | 29900 | 81.8 | 14900 | 7.80 | 54.8 | 103 | 373 | 3.16 | 13.0 | 1480 | |
| **2** | 675 | 77000 | 64800 | 681 | 22400 | 9130 | 9.7 | 605 | 0.39 | 7.1 | 43.4 | 152 | 1.02 | 1.0 | 1480 | |
| **3** | 330 | 4350 | 20400 | 3180 | 26500 | 36300 | 19.3 | 2320 | 1.15 | 16.5 | 57.5 | 192 | 0.93 | 1.6 | 3010 | |
| **4** | 980 | 708 | 28200 | 1450 | 11200 | 31900 | 29.4 | 1430 | 0.77 | 20.8 | 33.9 | 133 | 0.45 | 0.9 | 1020 | |
| **5** | 690 | 5210 | 9450 | 4782 | 18600 | 43000 | 23.6 | 2840 | 1.58 | 15.1 | 26.9 | 76.6 | 0.94 | 1.4 | 1450 | |
| **6** | 945 | 916 | 11200 | 596 | 13100 | 60900 | 13.3 | 265 | 0.27 | 8.2 | 19.1 | 68.5 | 0.47 | 0.4 | 1060 | |
| **7** | 140 | 805 | 58800 | 3560 | 20900 | 46000 | 24.6 | 2700 | 2.85 | 16.9 | 37.1 | 141 | 0.96 | 1.8 | 7270 | |
| **8** | 650 | 12900 | 1500 | 3330 | 11200 | 28800 | 75.3 | 18600 | 7.51 | 45.2 | 82.8 | 380 | 6.39 | 8.7 | 1540 | |
| **9** | 170 | 3630 | 2120 | 1360 | 20200 | 32200 | 32.0 | 1250 | 0.54 | 18.4 | 58.3 | 265 | 0.67 | 1.4 | 5850 | |
|  |  |  |  |  |  |  |  |  |  |  |  |  |  |  |  | |
|  |  |  |  |  |  |  |  |  |  |  |  |  |  |  |  | |
| ***purification*** | **Na** | **Mg** | **Al** | **P** | **Ca** | **Cr** | **Fe** | **Co** | **Ni** | **Cu** | **Zn** | **Ge** | **As** | **Se** |  | |
| **Sample** | **[µg L^-1^]** | **[µg L^-1^]** | **[µg L^-1^]** | **[µg L^-1^]** | **[µg L^-1^]** | **[µg L^-1^]** | **[µg L^-1^]** | **[µg L^-1^]** | **[µg L^-1^]** | **[µg L^-1^]** | **[µg L^-1^]** | **[µg L^-1^]** | **[µg L^-1^]** | **[µg L^-1^]** |  | |
| **AMScI** | 32500 | 5.8 | 7.4 | n/a | 87.0 | 1.51 | 22.9 | 0.03 | 0.66 | 0.4 | 6.7 | 0.01 | 0.09 | 0.5 |  | |
| **AMScII** | 26600 | 5.7 | 6.2 | n/a | 69.0 | 1.63 | 36.2 | 0.07 | 0.73 | 0.5 | 5.6 | <0.001 | 0.08 | 1.1 |  | |
| **AMScIII** | 30300 | 6.5 | 7.6 | n/a | 68.8 | 2.34 | 31.2 | 0.04 | 1.42 | 0.5 | 11.6 | 0.07 | 0.10 | 0.5 |  | |
| **AMScIV** | 28900 | 6.3 | 8.3 | n/a | 46.8 | 1.38 | 23.6 | 0.04 | 0.52 | 0.5 | 17.5 | <0.001 | 0.05 | 0.8 |  | |
| **AMSe1** | 8290 | 8100 | 8050 | n/a | 8140 | 6540 | 7480 | 7790 | 7910 | 7840 | 6230 | 58.3 | 46.3 | 58.6 |  | |
| **AMSe2** | 8320 | 8150 | 8060 | n/a | 8510 | 6620 | 7220 | 7940 | 8110 | 7950 | 6870 | 157 | 64.7 | 112 |  | |
| **AMSe3** | 8400 | 8450 | 8340 | n/a | 8570 | 6750 | 7100 | 8130 | 8220 | 8130 | 7130 | 240 | 67.1 | 144 |  | |
| **AMSe4** | 8460 | 8400 | 8330 | n/a | 9370 | 7060 | 7930 | 8220 | 8310 | 8250 | 6420 | 25.1 | 89.5 | 33.4 |  | |
| **AMSe5** | 8020 | 7830 | 7780 | n/a | 8230 | 6470 | 7250 | 7720 | 7810 | 7760 | 5900 | 71.8 | 49.2 | 57.8 |  | |
| **AMSe6** | 7990 | 7870 | 7860 | n/a | 8960 | 6310 | 5940 | 7740 | 7840 | 7740 | 6520 | 170 | 24.2 | 79.8 |  | |
| **AMSe7** | 7990 | 7890 | 7850 | n/a | 8460 | 6300 | 6600 | 7620 | 7720 | 7600 | 6050 | 78.6 | 33.6 | 97.0 |  | |
| **AMSe8** | 8100 | 7970 | 7920 | n/a | 8800 | 6720 | 7570 | 7740 | 7880 | 7810 | 5070 | 2.85 | 62.9 | 42.3 |  | |
| **AMSe9** | 77.9 | 5.0 | 12.0 | n/a | 61.8 | 0.15 | 4.9 | <0.01 | <0.01 | <0.08 | 3.7 | 0.20 | <0.02 | 3.2 |  | |
| **(continuation of Table D)** | | | | | | | | | | | | | | | |  |
|  | **Na** | **Mg** | **Al** | **P** | **Ca** | **Cr** | **Fe** | **Co** | **Ni** | **Cu** | **Zn** | **Ge** | **As** | **Se** |  | |
| **Sample** | **[µg L^-1^]** | **[µg L^-1^]** | **[µg L^-1^]** | **[µg L^-1^]** | **[µg L^-1^]** | **[µg L^-1^]** | **[µg L^-1^]** | **[µg L^-1^]** | **[µg L^-1^]** | **[µg L^-1^]** | **[µg L^-1^]** | **[µg L^-1^]** | **[µg L^-1^]** | **[µg L^-1^]** |  | |
| **AMSe**Blank | 82.0 | 4.5 | 14.1 | n/a | 59.6 | 4.95 | 1530 | 0.16 | 1.15 | 3.5 | 5.2 | 2.53 | <0.02 | 1.8 |  | |
| **AMSw1** | 1850 | 1730 | 1800 | n/a | 2020 | 1560 | 2250 | 1810 | 1850 | 1860 | 3930 | 0.08 | 683 | 340 |  | |
| **AMSw2** | 1590 | 1550 | 1620 | n/a | 1760 | 1470 | 2180 | 1560 | 1610 | 1630 | 3170 | 0.07 | 627 | 287 |  | |
| **AMSw3** | 1510 | 1510 | 1580 | n/a | 1630 | 1440 | 2350 | 1480 | 1520 | 1540 | 2900 | 0.07 | 726 | 348 |  | |
| **AMSw4** | 1610 | 1590 | 1650 | n/a | 1720 | 1380 | 1990 | 1560 | 1610 | 1620 | 3850 | 0.05 | 795 | 402 |  | |
| **AMSw5** | 2100 | 2110 | 2180 | n/a | 2280 | 1800 | 2560 | 2050 | 2110 | 2120 | 4380 | 0.32 | 884 | 305 |  | |
| **AMSw6** | 1730 | 1720 | 1780 | n/a | 1860 | 1520 | 2750 | 1670 | 1720 | 1740 | 3280 | 0.32 | 701 | 252 |  | |
| **AMSw7** | 1870 | 1890 | 1990 | n/a | 2010 | 1680 | 2550 | 1800 | 1870 | 1890 | 3770 | 0.05 | 689 | 252 |  | |
| **AMSw8** | 1990 | 1940 | 2010 | n/a | 2190 | 1670 | 2020 | 1890 | 1960 | 1970 | 5110 | 0.21 | 423 | 192 |  | |
| **AMSw9** | 31.1 | 3.1 | 5.3 | n/a | 93.9 | 0.23 | 6.6 | <0.01 | 0.16 | 0.2 | 6.5 | 0.40 | <0.02 | 7.0 |  | |
| **AMSw**Blank | 68.7 | 4.1 | 32.4 | n/a | 79.1 | 0.36 | 8.7 | <0.01 | 0.18 | 0.2 | 4.7 | <0.001 | <0.02 | 0.2 |  | |
| **AMSSe1** | 96.9 | 6.1 | 13.6 | n/a | 87.3 | 1051 | 24.3 | <0.01 | 0.60 | 1.5 | 5.2 | <0.001 | 2.40 | 428 |  | |
| **AMSSe2** | 135 | 9.4 | 27.3 | n/a | 104 | 993 | 22.4 | <0.01 | 0.60 | 1.7 | 4.8 | 0.03 | 4.10 | 377 |  | |
| **AMSSe3** | 116 | 6.0 | 21.4 | n/a | 82.9 | 959 | 30.1 | <0.01 | 0.45 | 1.5 | 11.7 | <0.001 | 2.40 | 458 |  | |
| **AMSSe4** | 116 | 6.4 | 11.5 | n/a | 66.0 | 986 | 20.9 | <0.01 | 0.30 | 1.5 | 4.1 | <0.001 | 2.00 | 459 |  | |
| **AMSSe5** | 218 | 7.1 | 13.4 | n/a | 89.0 | 981 | 29.1 | <0.01 | 0.60 | 1.8 | 5.6 | <0.001 | 4.15 | 427 |  | |
| **AMSSe6** | 113 | 6.7 | 18.4 | n/a | 81.4 | 1050 | 241 | <0.01 | 0.45 | 1.4 | 4.8 | <0.001 | 14.2 | 332 |  | |
| **AMSSe7** | 207 | 11.6 | 33.1 | n/a | 263 | 1700 | 67.3 | 0.39 | 0.80 | 2.2 | <0.1 | <0.001 | 6.00 | 717 |  | |
| **AMSSe8** | 105 | 8.2 | 12.8 | n/a | 125 | 803 | 24.3 | <0.01 | <0.01 | 8.4 | 7.6 | <0.001 | 0.85 | 413 |  | |
| **AMSSe9** | 137 | 10.9 | 16.6 | n/a | 125 | 1.05 | 45.0 | 0.12 | <0.01 | 1.2 | 12.1 | 0.03 | <0.02 | 0.2 |  | |
| **AMSSe**Blank | 83.5 | 6.6 | 12.3 | n/a | 90 | 0.55 | 25.3 | <0.01 | <0.01 | <0.08 | 4.8 | <0.001 | <0.02 | 0.2 |  | |
| **APPc1** | 5040 | 10.4 | 8.2 | 41.9 | 258 | 0.89 | 11.0 | 0.02 | 0.71 | 5.0 | 72.8 | 0.02 | 0.59 | 1.2 |  | |
| **APPc2** | 4790 | 11.8 | 8.1 | 30.5 | 251 | 1.06 | 10.5 | 0.05 | 2.48 | 11.9 | 143 | 0.02 | 0.62 | 0.3 |  | |
| **APPe1** | 776 | 478 | 916 | 587 | 1400 | 4.03 | 621 | 0.38 | 2.81 | 5.0 | 45.8 | 0.33 | 0.59 | 19.3 |  | |
| **APPe2** | 330 | 291 | 31.5 | <4.2 | 416 | 0.30 | 1.5 | 0.02 | 0.41 | 0.6 | 17.4 | <0.001 | <0.01 | 0.2 |  | |
| **APPe3** | 107 | 453 | 73.3 | 550 | 800 | 0.74 | 50.6 | 0.03 | 0.50 | 1.6 | 7.7 | <0.001 | 0.05 | 18.3 |  | |
| **APPe4** | 91.4 | 2140 | 111 | 796 | 2370 | 2.26 | 99.5 | 0.06 | 1.88 | 3.0 | 27.1 | <0.001 | 0.07 | 42.9 |  | |
| **APPe5** | 218 | 380 | 194 | 520 | 1650 | 1.03 | 106.2 | 0.06 | 1.24 | 1.5 | 11.6 | <0.001 | 0.07 | 20.6 |  | |
| **APPe6** | 85.1 | 845 | 47.3 | 203 | 4320 | 1.05 | 11.4 | 0.02 | 0.83 | 1.8 | 22.4 | <0.001 | <0.01 | 18.4 |  | |
| **(continuation of Table D)** | | | | | | | | | | | | | | | |  |
|  | **Na** | **Mg** | **Al** | **P** | **Ca** | **Cr** | **Fe** | **Co** | **Ni** | **Cu** | **Zn** | **Ge** | **As** | **Se** |  | |
| **Sample** | **[µg L^-1^]** | **[µg L^-1^]** | **[µg L^-1^]** | **[µg L^-1^]** | **[µg L^-1^]** | **[µg L^-1^]** | **[µg L^-1^]** | **[µg L^-1^]** | **[µg L^-1^]** | **[µg L^-1^]** | **[µg L^-1^]** | **[µg L^-1^]** | **[µg L^-1^]** | **[µg L^-1^]** |  | |
| **APPe7** | 24.5 | 676 | 45.0 | 192 | 4870 | 4.06 | 57.7 | 0.05 | 0.50 | 0.9 | 10.5 | <0.001 | <0.01 | 24.2 |  | |
| **APPe8** | 680 | 760 | 1560 | 79.0 | 1410 | 1.49 | 186 | 0.4 | 3.27 | 4.8 | 30.4 | 0.64 | 0.08 | 0.6 |  | |
| **APPe9** | 66.5 | 285 | 10.4 | 11.0 | 468 | 0.30 | 1.9 | 0.01 | 0.46 | 0.6 | 36.7 | <0.001 | <0.01 | <0.1 |  | |
| **APPe**Blank | 16.5 | 2.3 | 1.5 | 4.2 | 49.3 | 0.26 | 2.4 | <0.01 | 0.15 | 0.1 | 12.7 | <0.001 | <0.01 | <0.1 |  | |
| **APPw1** | 215 | 194 | 248 | 525 | 422 | 1.46 | 307 | 0.11 | 0.98 | 2.0 | 24.1 | 0.62 | 0.62 | 11.5 |  | |
| **APPw2** | 118 | 87.1 | 20.4 | 1680 | 179 | 0.37 | 35.7 | 0.01 | 0.32 | 2.3 | 28.9 | 0.53 | 0.10 | 52.9 |  | |
| **APPw3** | 49.0 | 126 | 21.0 | 476 | 286 | 0.42 | 28.9 | 0.01 | 0.34 | 1.2 | 54.9 | <0.001 | <0.01 | 17.3 |  | |
| **APPw4** | 36.1 | 507 | 27.5 | 489 | 611 | 0.71 | 35.8 | 0.02 | 0.68 | 1.0 | 32.3 | <0.001 | <0.01 | 25.9 |  | |
| **APPw5** | 78.8 | 119 | 58.4 | 625 | 557 | 0.45 | 57.7 | 0.03 | 0.41 | 1.6 | 19.2 | <0.001 | 0.07 | 10.2 |  | |
| **APPw6** | 44.8 | 228 | 14.0 | 1196 | 1150 | 0.40 | 16.8 | 0.01 | 0.44 | 0.7 | 29.2 | <0.001 | <0.01 | 18.5 |  | |
| **APPw7** | 23.9 | 168 | 11.8 | 139 | 1205 | 0.14 | 13.7 | 0.01 | 0.25 | 0.3 | 16.6 | <0.001 | <0.01 | 15.8 |  | |
| **APPw8** | 180 | 182 | 436 | 652 | 367 | 1.47 | 946 | 0.10 | 0.77 | 1.8 | 42.4 | <0.001 | 0.46 | 5.0 |  | |
| **APPw9** | 41.0 | 78.0 | 14.8 | 377 | 168 | 0.6 | 24.6 | <0.01 | 0.35 | 0.9 | 11.6 | <0.001 | 0.04 | 43.0 |  | |
| **APPw**Blank | 14.6 | 2.0 | 4.9 | <4.2 | 52.6 | 0.12 | 4.1 | <0.01 | 0.20 | 0.2 | 7.2 | <0.001 | <0.01 | <0.1 |  | |
| **APPSe1** | 14.6 | 5.5 | 24.1 | <4.2 | 62.3 | 0.83 | 13.8 | <0.01 | 0.29 | 0.2 | 4.3 | <0.001 | 0.10 | 61.3 |  | |
| **APPSe2** | 18.0 | 5.4 | 4.8 | <4.2 | 61.3 | 0.35 | 4.6 | <0.01 | 0.22 | 0.6 | 15.0 | <0.001 | 0.08 | 15.3 |  | |
| **APPSe3** | 35.9 | 6.1 | 6.3 | 13.8 | 143 | 0.28 | 5.5 | <0.01 | 0.40 | 0.5 | 40.1 | <0.001 | <0.01 | 16.1 |  | |
| **APPSe4** | 10.7 | 6.6 | 5.2 | <4.2 | 58.1 | 0.41 | 4.3 | <0.01 | 0.17 | 0.2 | 4.6 | <0.001 | 0.08 | 16.1 |  | |
| **APPSe5** | 12.0 | 5.5 | 10.6 | <4.2 | 81.1 | 0.34 | 6.0 | <0.01 | 0.16 | 0.3 | 6.6 | <0.001 | 0.01 | 31.5 |  | |
| **APPSe6** | 11.4 | 6.9 | 4.5 | 12.38 | 136 | 0.59 | 5.4 | <0.01 | 0.14 | 0.2 | 4.0 | <0.001 | 0.11 | 28.7 |  | |
| **APPSe7** | 17.0 | 5.0 | 5.1 | <4.2 | 134 | 0.42 | 5.9 | <0.01 | 0.24 | 0.2 | 12.2 | <0.001 | 0.01 | 72.9 |  | |
| **APPSe8** | 13.7 | 5.0 | 58.9 | 68.1 | 73.1 | 0.91 | 59.4 | <0.01 | 0.22 | 0.3 | 7.6 | <0.001 | 0.06 | 49.8 |  | |
| **APPSe9** | 160 | 7.4 | 13.9 | 11.3 | 144 | 0.94 | 6.5 | 0.02 | 0.63 | 0.8 | 15.5 | <0.001 | 0.14 | 20.9 |  | |
| **APPSe**Blank | 125 | 1.9 | 2.8 | <4.2 | 45.5 | 0.37 | 3.5 | <0.01 | 0.18 | 0.3 | 5.5 | 0.06 | 0.06 | <0.1 |  | |
|  | | | | | | | | | | | | | | | | |
| **Ubiquitary anions in samples before addition to method (A) column** (*milliequivalent absolute in sample (10 mL volumes)) | | | | | | | | | | | | | | | | |
| **Anion** | **Cl^-^** | | **NO_3_^-^** | | **PO_4_^3-^** | | **SO_4_^2-^** | |  |  |  |  |  |  |  | |
| **Sample** | **mg L^-1^** | **meq*** | **mg L^-1^** | **meq*** | **mg L^-1^** | **meq*** | **mg L^-1^** | **meq*** |  |  |  |  |  |  |  | |
| **1** | n.a. | - | 969 | 0.16 | 2.46 | 0.0008 | 10.6 | 0.0027 |  |  |  |  |  |  |  | |
| **2** | 2.60 | 0.0007 | 1.88 | 0.0003 | n.a. | - | 1.23 | 0.0003 |  |  |  |  |  |  |  | |

**Table E:** Matrix element and Se concentrations measured in multi-element standard and plants from Punjab for each step of method (B) purification

|  | **Na** | **Mg** | **Al** | **P** | **Ca** | **Cr** | **Fe** | **Co** | **Ni** | **Cu** | **Zn** | **Ge** | **As** | **Se** |
| --- | --- | --- | --- | --- | --- | --- | --- | --- | --- | --- | --- | --- | --- | --- |
| **Sample** | **[µg L^-1^]** | **[µg L^-1^]** | **[µg L^-1^]** | **[µg L^-1^]** | **[µg L^-1^]** | **[µg L^-1^]** | **[µg L^-1^]** | **[µg L^-1^]** | **[µg L^-1^]** | **[µg L^-1^]** | **[µg L^-1^]** | **[µg L^-1^]** | **[µg L^-1^]** | **[µg L^-1^]** |
| **BMScI** | 38.4 | 5.9 | 7.8 | n/a | 345 | 0.83 | 7.3 | <0.01 | 0.56 | 0.4 | 16.5 | 0.13 | 0.14 | 1.5 |
| **BMScII** | 73.2 | 9.0 | 18.8 | n/a | 399 | 0.63 | 12.1 | <0.01 | 0.77 | 0.7 | 18.6 | <0.001 | 0.34 | 2.6 |
| **BMSe3** | 9850 | 9660 | 9500 | n/a | 9680 | 9680 | 9690 | 9920 | 9670 | 4840 | 9770 | 0.25 | 348 | 19.4 |
| **BMSe4** | 9720 | 9800 | 9680 | n/a | 9640 | 9580 | 9590 | 9680 | 9510 | 3820 | 9580 | 0.18 | 570 | 7.4 |
| **BMSe**Blank | 30.8 | 5.2 | 9.2 | n/a | 141 | 0.72 | 16.1 | 0.04 | 0.30 | 0.6 | 7.9 | <0.001 | 0.23 | <0.1 |
| **BMSe6** | 9950 | 10200 | 10100 | n/a | 9800 | 9680 | 9660 | 9840 | 9630 | 3670 | 9730 | 0.20 | 1610 | 7.7 |
| **BMSe7** | 9820 | 10100 | 9970 | n/a | 9640 | 9590 | 9600 | 9730 | 9610 | 1210 | 9700 | 0.15 | 1741 | 33.1 |
| **BMSe8** | 9020 | 94000 | 9240 | n/a | 9140 | 8800 | 8790 | 8970 | 8870 | 692 | 8980 | 0.22 | 1080 | 5.8 |
| **BMSe9** | 9570 | 9920 | 9860 | n/a | 10300 | 9370 | 9380 | 9500 | 9440 | 910 | 9450 | 0.12 | 1970 | 8.6 |
| **BMSe10** | 4890 | 4650 | 4580 | n/a | 4920 | 4860 | 4970 | 4860 | 4860 | 2080 | 4890 | 0.14 | 339 | 12.0 |
| **BMSw3** | 190 | 188 | 184 | n/a | 231 | 184 | 192 | 185 | 183 | 4790 | 192 | <0.001 | 0.35 | 2.6 |
| **BMSw4** | 243 | 211 | 230 | n/a | 851 | 199 | 250 | 202 | 198 | 5490 | 260 | 0.08 | 0.76 | 2.3 |
| **BMSw**Blank | 72.5 | 5.1 | 48.5 | n/a | 69.2 | 0.22 | 10.5 | <0.01 | 0.35 | 0.8 | 4.2 | <0.001 | 0.32 | <0.1 |
| **BMSw6** | 229 | 231 | 247 | n/a | 307 | 221 | 228 | 224 | 225 | 5860 | 234 | 0.11 | 2.45 | 2.9 |
| **BMSw7** | 267 | 264 | 256 | n/a | 383 | 255 | 262 | 256 | 260 | 7860 | 267 | <0.001 | 4.23 | 5.8 |
| **BMSw8** | 253 | 248 | 250 | n/a | 400 | 237 | 240 | 237 | 241 | 7960 | 248 | 0.01 | 10.2 | 5.1 |
| **BMSw9** | 412 | 339 | 336 | n/a | 523 | 326 | 339 | 330 | 336 | 8280 | 345 | 0.01 | 5.66 | 4.3 |
| **BMSw10** | 109 | 99.7 | 101 | n/a | 206 | 99.4 | 108 | 98.7 | 98.4 | 2740 | 106 | <0.001 | 0.34 | 3.5 |
| **BMSSe3** | 67.2 | 26.8 | 35.1 | n/a | 643 | 3.56 | 18.1 | 0.22 | 1.97 | 131 | 12.6 | 1.03 | 27.0 | 681 |
| **BMSSe4** | 93.6 | 24.6 | 28.6 | n/a | 1140 | 0.37 | 8.7 | 0.09 | 0.49 | 381 | 24.5 | 1.17 | 53.5 | 201 |
| **BMSSe**Blank | 131 | 24.3 | 27.4 | n/a | 1410 | 0.30 | 7.9 | <0.01 | 0.30 | 0.8 | 30.4 | 0.08 | 0.51 | <0.1 |
| **BMSSe6** | 110 | 25.0 | 34.5 | n/a | 989 | 1.11 | 16.0 | 0.22 | 1.04 | 344 | 32.6 | 2.62 | 164 | 1420 |
| **BMSSe7** | 98.0 | 22.2 | 26.9 | n/a | 827 | 0.60 | 13.2 | 0.12 | 0.72 | 959 | 18.5 | 7.61 | 389 | 2990 |
| **BMSSe8** | 92.5 | 22.5 | 34.7 | n/a | 829 | 0.66 | 12.2 | 0.15 | 0.69 | 756 | 21.8 | 13.0 | 569 | 2210 |
| **BMSSe9** | 106 | 24.5 | 30.0 | n/a | 970 | 0.80 | 9.2 | 0.15 | 0.72 | 1010 | 24.8 | 28.9 | 695 | 4510 |
| **BMSSe10** | 90.4 | 24.6 | 60.3 | n/a | 954 | 0.46 | 11.3 | 0.06 | 0.44 | 121 | 20.7 | 1.69 | 34.2 | 881 |
| **BPPc1** | 212 | 16.4 | 14.4 | 22.3 | 325 | 0.33 | 13.4 | 0.02 | 0.77 | 0.4 | 11.3 | 1.38 | 0.19 | 1.3 |
| **BPPc2** | 130 | 7.1 | 3.8 | 14.8 | 152 | 0.27 | 4.7 | <0.01 | 0.37 | 0.4 | 5.4 | 0.01 | 0.26 | 1.0 |
| **(continuation of Table E)** | | | | | | | | | | | | | | |
|  | **Na** | **Mg** | **Al** | **P** | **Ca** | **Cr** | **Fe** | **Co** | **Ni** | **Cu** | **Zn** | **Ge** | **As** | **Se** |
| **Sample** | **[µg L^-1^]** | **[µg L^-1^]** | **[µg L^-1^]** | **[µg L^-1^]** | **[µg L^-1^]** | **[µg L^-1^]** | **[µg L^-1^]** | **[µg L^-1^]** | **[µg L^-1^]** | **[µg L^-1^]** | **[µg L^-1^]** | **[µg L^-1^]** | **[µg L^-1^]** | **[µg L^-1^]** |
| **BPPe1** | 1140 | 963 | 1250 | 962 | 2010 | 5.79 | 906 | 0.55 | 4.81 | 7.5 | 44.2 | 0.22 | 0.97 | 16.8 |
| **BPPe2** | 477 | 402 | 47.5 | 1440 | 619 | 1.15 | 45.9 | 0.05 | 0.99 | 4.0 | 19.4 | 0.12 | 0.10 | 3.6 |
| **BPPe3** | 112 | 599 | 98.0 | 764 | 1050 | 0.77 | 91.4 | 0.05 | 0.74 | 2.4 | 11.1 | 0.35 | 0.04 | 1.9 |
| **BPPe4** | 46.0 | 2320 | 126 | 1030 | 2650 | 3.12 | 138 | 0.09 | 2.15 | 3.8 | 17.8 | 0.02 | 0.09 | 4.2 |
| **BPPe5** | 344 | 572 | 276 | 1270 | 2570 | 1.79 | 205 | 0.12 | 1.35 | 2.5 | 15.7 | 0.24 | 0.11 | 8.1 |
| **BPPe6** | 107 | 959 | 58.8 | 1130 | 5010 | 1.48 | 29.1 | 0.03 | 1.26 | 2.7 | 13.9 | 0.28 | 0.11 | 2.0 |
| **BPPe7** | 7.2 | 716 | 45.1 | 250 | 5240 | 0.57 | 45.7 | 0.05 | 1.01 | 1.3 | 5.9 | 0.09 | 0.05 | 2.5 |
| **BPPe8** | 824 | 832 | 1750 | 724 | 1580 | 5.28 | 1160 | 0.50 | 3.22 | 6.1 | 35.5 | 0.46 | 0.59 | 3.0 |
| **BPPe9** | 49.8 | 314 | 25.7 | 302 | 529 | 0.73 | 27.2 | 0.02 | 0.57 | 1.4 | 10.5 | 0.85 | 0.03 | 2.0 |
| **BPPe**Blank | 25.2 | 3.6 | 4.4 | 5.2 | 97.2 | 0.23 | 4.7 | <0.01 | 0.31 | 0.4 | 4.6 | 0.09 | 0.05 | <0.1 |
| **BPPw1** | 16.3 | 27.3 | 43.4 | 26.0 | 92.3 | 0.32 | 25.4 | 0.04 | 0.21 | 0.4 | 4.2 | 0.26 | 0.02 | 1.0 |
| **BPPw2** | 4.8 | 12.7 | 16.0 | 50.6 | 45.5 | 0.14 | 3.1 | <0.01 | 0.08 | 0.3 | 3.4 | 0.01 | 0.05 | 1.4 |
| **BPPw3** | 37.5 | 21.6 | 8.1 | 25.1 | 143 | 0.21 | 8.2 | 0.02 | 0.85 | 5.2 | 40.4 | 0.46 | 0.31 | 0.6 |
| **BPPw4** | 6.7 | 71.2 | 7.5 | 37.9 | 106 | 0.21 | 7.8 | <0.01 | 0.33 | 0.3 | 2.7 | <0.001 | 0.02 | 0.8 |
| **BPPw5** | 4.3 | 20.8 | 10.3 | 37.1 | 132 | 0.49 | 9.3 | <0.01 | 0.60 | 0.2 | 3.7 | 0.02 | 0.02 | 0.5 |
| **BPPw6** | 33.1 | 31.4 | 7.5 | 44.8 | 270 | 0.66 | 7.9 | 0.02 | 0.95 | 3.9 | 32.4 | 0.21 | 0.24 | 0.8 |
| **BPPw7** | 9.4 | 23.6 | 5.9 | 7.7 | 191 | 0.28 | 5.0 | <0.01 | 0.08 | 0.3 | 3.4 | 0.01 | 0.07 | 1.1 |
| **BPPw8** | 9.7 | 25.3 | 54.3 | 23.2 | 67.7 | 0.28 | 36.3 | 0.02 | 0.15 | 0.3 | 2.8 | 0.02 | 0.03 | 0.8 |
| **BPPw9** | 9.3 | 10.3 | 2.9 | 12.8 | 38.0 | 0.27 | 3.6 | <0.01 | 0.12 | 0.3 | 2.5 | <0.001 | 0.02 | 0.6 |
| **BPPw**Blank | 17.3 | 3.9 | 3.9 | 1.6 | 95.4 | 0.18 | 3.8 | <0.01 | 0.68 | 2.9 | 27.5 | 0.03 | 0.15 | 0.1 |
| **BS1** | 9.2 | 5.4 | 3.9 | 3.5 | 291 | 0.13 | 3.4 | 0.02 | 0.36 | 1.0 | 31.4 | <0.001 | 0.22 | 1.5 |
| **BS2** | 17.1 | 6.0 | 5.1 | 6.6 | 363 | 0.09 | 3.3 | 0.06 | 0.19 | 0.2 | 32.3 | 0.03 | 0.09 | 1.5 |
| **BPPSe1** | 23.1 | 28.0 | 19.4 | 45.4 | 434 | 0.36 | 15.2 | 0.03 | 0.39 | 0.7 | 98.2 | 0.06 | 0.16 | 40.2 |
| **BPPSe2** | 42.2 | 24.8 | 17.2 | 28.6 | 766 | 0.23 | 18.1 | <0.01 | 0.27 | 0.9 | 96.2 | 0.08 | 0.18 | 96.2 |
| **BPPSe3** | 26.7 | 20.5 | 15.7 | 45.7 | 403 | 0.85 | 21.4 | <0.01 | 0.25 | 0.6 | 63.3 | 0.02 | 0.15 | 77.1 |
| **BPPSe4** | 20.8 | 18.7 | 12.4 | 23.1 | 334 | 0.20 | 14.6 | <0.01 | 0.37 | 1.0 | 67.6 | 0.03 | 0.26 | 28.5 |
| **BPPSe5** | 27.4 | 18.4 | 18.7 | 15.6 | 324 | 0.24 | 13.1 | <0.01 | 0.13 | 0.7 | 6.1 | 0.04 | 0.33 | 20.8 |
| **BPPSe6** | 29.1 | 18.3 | 16.1 | 21.9 | 331 | 0.30 | 16.7 | <0.01 | 0.16 | 0.6 | 8.7 | 0.05 | 0.52 | 50.7 |
| **BPPSe7** | 22.4 | 21.8 | 23.0 | 23.7 | 314 | 0.18 | 50.3 | <0.01 | 1.88 | 3.4 | 59.1 | 0.01 | 0.25 | 86.0 |
| **BPPSe8** | 19.7 | 18.4 | 13.0 | 12.4 | 284 | 0.37 | 12.2 | <0.01 | 0.23 | 0.5 | 81.3 | 0.40 | 0.30 | 52.8 |
| **BPPSe9** | 29.5 | 19.7 | 13.6 | 28.0 | 272 | 1.03 | 29.8 | <0.01 | 0.45 | 0.7 | 75.7 | 0.02 | 0.27 | 94.4 |
| **BPPSe**Blank | 50.4 | 23.4 | 15.2 | 28.0 | 426 | 0.34 | 17.2 | <0.01 | 0.39 | 0.8 | 86.6 | 0.14 | 0.20 | <0.1 |

**Table F:** Matrix element and Se concentrations measured in multi-element standard for each step of method (C) purification (Na was not determined due to 1M NaOH disturbances being by far higher than sample Na concentrations)

|  | **Mg** | **Al** | **Ca** | **Cr** | **Fe** | **Co** | **Ni** | **Cu** | **Zn** | **Ge** | **As** | **Se** |
| --- | --- | --- | --- | --- | --- | --- | --- | --- | --- | --- | --- | --- |
| **Sample** | **[µg L^-1^]** | **[µg L^-1^]** | **[µg L^-1^]** | **[µg L^-1^]** | **[µg L^-1^]** | **[µg L^-1^]** | **[µg L^-1^]** | **[µg L^-1^]** | **[µg L^-1^]** | **[µg L^-1^]** | **[µg L^-1^]** | **[µg L^-1^]** |
| **iMS1** | 16200 | 16700 | 16200 | 16400 | 17200 | 16500 | 16200 | 16500 | 16200 | 1530 | 1540 | 1620 |
| **iMS2** | 16300 | 16500 | 16800 | 16100 | 16800 | 16200 | 15800 | 16200 | 16000 | 1470 | 1520 | 1510 |
| **HGMS1** | 8.0 | 20.5 | 288 | 9.26 | 39.6 | <0.006 | 38.9 | <0.1 | 10.8 | <0.006 | <0.02 | <0.1 |
| **HGMS2** | 10.9 | 27.5 | 378 | 9.00 | 41.0 | <0.006 | 39.1 | <0.1 | 13.3 | <0.006 | <0.02 | <0.1 |
| **AEMSe1** | 10.7 | 37.5 | 370 | 1.80 | 47.6 | <0.006 | <0.08 | <0.1 | 15.9 | <0.006 | <0.02 | <0.1 |
| **AEMSe2** | 10.7 | 29.0 | 365 | 0.48 | 42.8 | <0.006 | <0.08 | <0.1 | 19.6 | <0.006 | <0.02 | <0.1 |
| **AEMSw1** | 7.1 | 9.0 | 110 | 0.18 | 10.7 | 0.02 | 0.4 | 0.4 | 3.8 | 0.02 | 0.20 | 0.4 |
| **AEMSw2** | 3.6 | 6.2 | 77.8 | 0.20 | 8.8 | 0.03 | 0.4 | 0.3 | 2.9 | 0.03 | 0.06 | 0.3 |
| **AEMSSe1** | 3.2 | 3.1 | 58.7 | 11.4 | 1.8 | 0.12 | 37.9 | 0.2 | 1.7 | <0.006 | 0.26 | 0.5 |
| **AEMSSe2** | 2.7 | 2.7 | 31.3 | 11.6 | 2.1 | 0.10 | 38.2 | 0.2 | 1.6 | <0.006 | 0.26 | 0.4 |
| **AEMSSe**Blank | 5.3 | 6.7 | 177 | 42.7 | 42.7 | 17.0 | 2.8 | 0.2 | 66.0 | 0.48 | 2.28 | <0.1 |

**Table G:** Se concentrations measured in purified samples of methods (A) (including modifications), (B) and (C) for all matrices used

| ***Modifications (performed with Punjab Plants (PP))*** | | | | |
| --- | --- | --- | --- | --- |
| **Se [µg L^-1^]** | Ellis et al. (2003) | Variation I | Variation II | Variation III |
| **PP1** | 118 | 43.8 | 66.5 | 92.2 |
| **PP2** | 30.0 | 21.3 | 208 | 96.6 |
| **PP3** | 31.2 | 83.1 | 24.5 | 99.8 |
| **PP4** | 31.0 | 19.2 | 68.6 | 134 |
| **Average (n=4)** | **52.5 ±32.6** | **41.9 ±21.6** | **91.8 ±58.0** | **106 ±14.0** |
|  |  |  |  |  |
| ***Se only (initial 1000 ng Se)*** | | | | |
| **Se [µg L^-1^]** | **(A)** | **(B)** | **(C) - after HG** | **(C) - after HG+AE** |
| Se_1 | 199 | 107 | 132 | n/a |
| Se_2 | 200 | 105 | 115 | n/a |
| Se_3 | 203 | 109 |  |  |
| **Average** | **50.1 ±0.4** | **107 ±1.6** | **124 ±8.3** |  |
|  |  |  |  |  |
| ***Se free cultivated plants (cp) + doped Se (1000 ng)*** | | | | |
| **Se [µg L^-1^]** | **(A)** | **(B)** | **(C) - after HG** | **(C) - after HG+AE** |
| Se_cp_1 | 193 | 114 | 138 | 21.6 |
| Se_cp_2 | 199 | 111 | 133 | 24.9 |
| Se_cp_3 | 203 | 110 | 166 | 50.4 |
| Se_cp_4 |  |  | 166 | 56.4 |
| Se_cp_5 |  |  | 125 | 118 |
| Se_cp_6 |  |  | 156 | 152 |
| Se_cp_7 |  |  | 131 | 39.3 |
| Se_cp_8 |  |  | 137 | 70.8 |
| Se_cp_9 |  |  | 139 | 106 |
| Se_cp_10 |  |  | 143 | 108 |
| **average** | **198 ±2.8** | **111 ±1.5** | **143 ±11.7** | **74.8 ±37.2** |
|  |  |  |  |  |
| ***Se free phytoagar (p) + doped Se (1000 ng)*** | | | | |
| **Se [µg L^-1^]** | **(A)** | **(B)** | **(C) - after HG** | **(C) - after HG+AE** |
| Se_p_1 | 131 | 114 | 159 | 94.4 |
| Se_p_2 | 115 | 116 | 149 | 86.7 |
| Se_p_3 | 48.4 | 114 |  |  |
| **average** | **98.2 ±24.9** | **115 ±1.2** | **154 ±4.9** | **90.5 ±3.9** |
|  |  |  |  |  |
| ***Reference materials used for validation (chapter 4.5.3) (WF-Wheat Flour NISTSRM1567a)*** | | | | |
| **Se [µg L^-1^]** | **(A)** | **(B)** | **(C) - after HG** | **(C) - after HG+AE** |
| WF1 | n/a | n/a | 161 | 114 |
| WF2 | n/a | n/a | 165 | 96.1 |
| SGR-1 | n/a | n/a | n/a | 45.1 |

**Table H:** TOC residuals measured in purified plant and phytoagar samples of methods (A), (B) and (C)

|  | **TOC in plant digests [mg L^-1^]** | | | **TOC in phytoagar extracts [mg L^-1^]** | | |
| --- | --- | --- | --- | --- | --- | --- |
| **purification method** | **A** | **B** | **C** | **A** | **B** | **C** |
| **I** | 10.5 | 12.9 | <0.9 | 52.8 | 57.3 | <0.9 |
| **II** | 7.4 | 20.1 | <0.9 | 121 | 80.5 | <0.9 |
| **III** | 5.6 | 26.8 | <0.9 | 45.2 | 11.8 | <0.9 |
| **average** | **7.8 ±1.8** | **20.0 ±4.7** | **<0.9** | **73.0 ±32.0** | **49.9 ±25.4** | **<0.9** |

**Table I:** Uncorrected and corrected Se isotope ratios, internal errors, instrumental mass bias and Se recoveries determined in internal reproducibility and validation test samples of methods (A) (GLS), (B) (CTR) and (C) (HGT) (correction = NIST subtraction) (*added before digestion **added after digestion, ^1)^ after HG and anion exchange

| **Purification method** | **Sample ID** | **matrix** | **DS addition** | **δ^82/76^Se [‰] (2SD filter)** | **internal error (2 SE)** | **δ^82^Se [‰]** | **β_instr_** | **Se recovery after HG [%]** | **total Se recovery [%]^1)^** |
| --- | --- | --- | --- | --- | --- | --- | --- | --- | --- |
| ***Internal Reproducibility*** | |  |  |  |  |  |  |  |  |
| **(B)** | Pl_I1 B | cultivated plant | ** | -1.91 | 0.08 | -1.37 | -2.13 | n/a | n/a |
|  | Pl_I1 B | cultivated plant | ** | -1.91 | 0.09 | -1.37 | -2.13 | n/a | n/a |
|  | Pl_I2 B | cultivated plant | ** | -1.86 | 0.08 | -1.33 | -2.14 | n/a | n/a |
|  | Pl_I2 B | cultivated plant | ** | -1.85 | 0.08 | -1.33 | -2.13 | n/a | n/a |
|  | Ag_I1 B | phytoagar | ** | -2.06 | 0.05 | -1.53 | -2.12 | n/a | n/a |
|  | Ag_I1 B | phytoagar | ** | -2.05 | 0.04 | -1.52 | -2.12 | n/a | n/a |
|  | Ag_I2 B | phytoagar | ** | -1.27 | 0.05 | -0.74 | -2.12 | n/a | n/a |
|  | Ag_I2 B | phytoagar | ** | -1.25 | 0.04 | -0.73 | -2.13 | n/a | n/a |
| ***Validation*** |  |  |  |  |  |  |  |  |  |
| **(A)** | Pl1 A | Se doped plant | ** | -0.77 | 0.03 | 0.42 | -1.98 | n/a | 96.3 |
|  | Pl2 A | Se doped plant | ** | 2.42 | 0.06 | 3.58 | -1.90 | n/a | 99.5 |
|  | Ag1 A | Se doped phytoagar | ** | 5.57 | 0.02 | 6.74 | -1.85 | n/a | 65.5 |
|  | Ag2 A | Se doped phytoagar | ** | 3.14 | 0.02 | 4.32 | -1.88 | n/a | 57.7 |
| **(B)** | Pl1 B | Se doped plant | * | 24.71 | 0.07 | 26.11 | -1.71 | n/a | 5.2 |
|  | Pl2 B | Se doped plant | * | 11.42 | 0.12 | 12.82 | -1.90 | n/a | 2.4 |
|  | Pl3 B | Se doped plant | * | 21.08 | 0.12 | 22.48 | -1.76 | n/a | 2.6 |
|  | | | | | | | | | |
| **Purification method** | **Sample ID** | **matrix** | **DS addition** | **δ^82/76^Se [‰] (2SD filter)** | **internal error (2 SE)** | **δ^82^Se [‰]** | **β_instr_** | **Se recovery after HG [%]** | **total Se recovery [%]^1)^** |
| **(B)** | Pl4 B | Se doped plant | * | 31.53 | 0.09 | 32.94 | -1.59 | n/a | 12.3 |
|  | Pl5 B | Se doped plant | * | 34.58 | 0.20 | 35.98 | -1.55 | n/a | 1.1 |
|  | Pl6 B | Se doped plant | * | 25.40 | 0.82 | 26.80 | -1.70 | n/a | 0.3 |
|  | Pl7 B | Se doped plant | ** | -0.57 | 0.16 | 0.86 | -1.88 | n/a | 1.7 |
|  | Pl8 B | Se doped plant | ** | -4.65 | 0.19 | -3.22 | -1.92 | n/a | 1.3 |
|  | Pl9 B | Se doped plant | ** | 4.99 | 0.05 | 6.40 | -1.80 | n/a | 25.5 |
|  | Pl10 B | Se doped plant | ** | 33.81 | 0.21 | 35.22 | -1.45 | n/a | 4.9 |
|  | Pl11 B | Se doped plant | ** | 17.91 | 0.04 | 19.31 | -1.74 | n/a | 29.4 |
|  | Pl12 B | Se doped plant | ** | 13.58 | 0.20 | 14.98 | -1.83 | n/a | 5.5 |
|  | Ag1 B | Se doped phytoagar | ** | 19.89 | 0.20 | 21.29 | -1.68 | n/a | 4.2 |
|  | Ag2 B | Se doped phytoagar | ** | 35.67 | 0.32 | 37.07 | -1.46 | n/a | 2.6 |
| **(C)** | Pl1 C | Se doped plant | * | -0.14 | 0.16 | 0.74 | -2.01 | 83.0 | 13.0 |
|  | Pl2 C | Se doped plant | * | 1.21 | 0.34 | 2.10 | -1.95 | 79.5 | 14.9 |
|  | Pl3 C | Se doped plant | ** | -1.00 | 0.03 | 0.00 | -2.04 | 79.5 | 14.9 |
|  | Pl3 C | Se doped plant | ** | -1.00 | 0.02 | 0.00 | -2.05 | 99.5 | 30.2 |
|  | Pl4 C | Se doped plant | ** | -0.98 | 0.02 | 0.02 | -2.07 | 99.9 | 33.9 |
|  | Pl5 C | Se doped plant | ** | -0.44 | 0.05 | 0.40 | -1.99 | 75.2 | 71.0 |
|  | Pl6 C | Se doped plant | ** | -1.09 | 0.05 | -0.24 | -2.02 | 93.7 | 91.3 |
|  | Pl7 C | Se doped plant | ** | -3.65 | 0.04 | 0.37 | -2.02 | 78.5 | 23.6 |
|  | Pl8 C | Se doped plant | ** | -3.71 | 0.03 | 0.34 | -2.02 | 81.9 | 42.5 |
|  | Pl9 C | Se doped plant | ** | -3.71 | 0.04 | 0.34 | -2.04 | 83.5 | 63.8 |
|  | Pl10 C | Se doped plant | ** | -3.70 | 0.05 | 0.33 | -2.04 | 85.6 | 64.9 |
|  | Ag1 C | Se doped phytoagar | ** | -3.25 | 0.04 | 1.04 | -2.01 | 95.1 | 56.6 |
|  | Ag1 C | Se doped phytoagar | ** | -3.25 | 0.05 | 1.04 | -2.00 | 95.1 | 56.6 |
|  | Ag2 C | Se doped phytoagar | ** | -3.27 | 0.07 | 1.13 | -2.01 | 89.3 | 52.0 |
|  | SGR-1 | SGR-1 | ** | -3.62 | 0.10 | 0.74 | -2.01 | n/a | 27.0 |
|  | WF1 | NISTSRM1567a | ** | -3.99 | 0.07 | 0.34 | -1.99 | 96.4 | 68.3 |
|  | WF2 | NISTSRM1567a | ** | -4.32 | 0.05 | 0.19 | -2.00 | 99.1 | 57.6 |

**Table J:** Se species determined in a plant digest (according to Bell et al. 1992), in the oxidized and reduced samples added to columns of methods (A) and (B) and their respective Se extracts as well as in phytoagar after cultivation (n.q.: not quantifiable though visible peak; - : no visible peak)

|  |  | **Ion Exchange (Bird et al. 1997)** | | | **Ion Pair (Bird et al. 1997)** | |  |
| --- | --- | --- | --- | --- | --- | --- | --- |
| **Sample** | **Details** | **selenate** | **selenite** | **unknown** | **Inorg.Se** | **Org. Se** | **Share organic** |
|  |  | **[ppb]** | **[ppb]** | **[ppb]** | [ppb] | [ppb] | [%] |
| **PP7** | **Plant digest (Bell et al., 1992)** | 311 | 116 | 427 | 183 | 34.1 | 15.7 |
| **AO1** | **Oxidized with K2S2O8** | 150 | - | 150 | n/a | n/a | n/a |
| **AO4** |  | 131 | - | 131 | n/a | n/a | n/a |
| **BR1** | **Reduced with 4M HCl** | - | 63.7 | <0.1 | n/a | n/a | n/a |
| **BR4** |  | - | 27.7 | <0.1 | n/a | n/a | n/a |
| **APPSe1** | **Method (A) Se extract** | 68.1 | - | 68.1 | n/a | n/a | n/a |
| **APPSe4** |  | 104 | - | 104.4 | n/a | n/a | n/a |
| **BPPSe1** | **Method (B) Se extract** | - | 135 | 134.7 | n/a | n/a | n/a |
| **BPPSe4** |  | - | 67.3 | 67.3 | n/a | n/a | n/a |
|  |  |  |  |  |  |  |  |
| **Sample** | **Species added** | **selenate** | **selenite** | **unknown** | **selenite** | **SeMet** | **total** |
|  |  | **[ppb]** | **[ppb]** | **[ppb]** | **[ppb]** | **[ppb]** | **[ppb]** |
| **IVpac1** | **selenate** | n.q. | - | - | - | - | 13.5 |
| **IVpac2** | **selenate** | 87.4 | - | 4.8 | - | - | 101 |
| **IVpac3** | **selenate** | 889 | - | - | - | - | 775 |
| **IVpac4** | **selenite** | - | 3.9 | 2.9 | n.q. | - | 36.4 |
| **IVpac5** | **selenite** | - | 85.9 | 12.4 | 70.1 | - | 216 |
| **IVpac6** | **selenite** | - | 104 | - | 105 | - | 190 |
| **IVpac7** | **SeMet** | - | - | 3.4 | 31.8 | 6.6 | 26.6 |
| **IVpac8** | **SeMet** | - | - | 12.0 | n.q. | n.q. | 94.5 |
| **IVpac9** | **SeMet** | - | - | 60.8 | - | 60.1 | 212 |
|  |  |  |  |  |  |  |  |
|  |  |  |  |  |  |  |  |
|  |  |  |  |  |  |  |  |
|  |  |  |  |  |  |  |  |
|  |  |  |  |  |  |  |  |
|  |  |  |  |  |  |  |  |
| **(continuation of Table J)** | | | | | | | |
| **Sample** | **Species added** | **selenate** | **selenite** | **unknown** | **selenite** | **SeMet** | **total** |
|  |  | **[ppb]** | **[ppb]** | **[ppb]** | **[ppb]** | **[ppb]** | **[ppb]** |
| **Vpac1** | **selenate** | n.q. | - | - | - | - | 8.8 |
| **Vpac2** | **selenate** | 136 | - | - | - | - | 154 |
| **Vpac3** | **selenate** | 743 | - | - | - | - | 679 |
| **Vpac4** | **selenite** | - | 15.1 | - | 2.2 | 23.3 | 33.4 |
| **Vpac5** | **selenite** | - | 14.9 | 14.3 | n.q. | - | 46.0 |
| **Vpac6** | **selenite** | - | 68.6 | - | 77.0 | - | 134 |
| **Vpac7** | **SeMet** | - | - | 2.9 | 1.9 | 0.7 | 21.9 |
| **Vpac8** | **SeMet** | - | - | 22.5 | n.q. | - | 82.4 |
| **Vpac9** | **SeMet** | - | - | 45.7 | 17.1 | 4.1 | 168 |

**Table K:** Se concentrations measured in plants (roots + shoots) and phytoagar after cultivation of MinPaX I-V (dig – plant digest, fil – phytoagar filtrate)

| **MinPaX I** | | | | **MinPaX V** | | | |  |  |  |  |
| --- | --- | --- | --- | --- | --- | --- | --- | --- | --- | --- | --- |
| **Sample ID** | **weight sample [mg]** | **Se in dig/fil [µg L^-1^]** | **Se in solid [ppm]** | **Sample ID** | **weight sample [mg]** | **Se in dig/fil [µg L^-1^]** | **Se in solid [ppm]** |  |  |  |  |
| **Ipac1** |  | 2.7 |  | **Vpac1** |  | 3.4 |  |  |  |  |  |
| **Ipac2** |  | 15.9 |  | **Vpac2** |  | 194 |  |  |  |  |  |
| **Ipac3** |  | 78.1 |  | **Vpac3** |  | 954 |  |  |  |  |  |
| **Ipac4** |  | 4.9 |  | **Vpac4** |  | 93.8 |  |  |  |  |  |
| **Ipac5** |  | 48.5 |  | **Vpac5** |  | 418 |  |  |  |  |  |
| **Ipac6** |  | 113 |  | **Vpac6** |  | 966 |  |  |  |  |  |
| **Ipac7** |  | 2.5 |  | **Vpac7** |  | 26.1 |  |  |  |  |  |
| **Ipac8** |  | 25.2 |  | **Vpac8** |  | 310 |  |  |  |  |  |
| **Ipac9** |  | 61.0 |  | **Vpac9** |  | 413 |  |  |  |  |  |
| **Ipac10** |  | <0.1 |  | **Vpac10** |  | <0.1 |  |  |  |  |  |
| **Icpr1** | 49 | 125 | 25.6 | **Vcpr1** | 55.9 | 28.7 | 1.60 |  |  |  |  |
| **Icpr2** | 52 | 619 | 119 | **Vcpr2** | 27.2 | 188 | 5.12 |  |  |  |  |
| **Icpr3** | 39 | 1370 | 357 | **Vcpr3** | 36.0 | 197 | 7.08 |  |  |  |  |
| **Icpr4** | 74 | 268 | 36.0 | **Vcpr4** | 13.3 | 32.9 | 0.44 |  |  |  |  |
| **Icpr5** | 125 | 1150 | 92.1 | **Vcpr5** | 51.3 | 128 | 6.56 |  |  |  |  |
| **Icpr6** | 62 | 857 | 139 | **Vcpr6** | 37.0 | 128 | 4.72 |  |  |  |  |
| **Icpr7** | 64 | 312 | 48.9 | **Vcpr7** | 63.1 | 52 | 3.26 |  |  |  |  |
| **Icpr8** | 87 | 1440 | 167 | **Vcpr8** | 24.0 | 381 | 9.14 |  |  |  |  |
| **Icpr9** | 63 | 2390 | 381 | **Vcpr9** | 44.4 | 734 | 32.6 |  |  |  |  |
| **Icpr10** | 72 | 213 | 29.8 | **Vcpr10** | 48.6 | 0.3 | 0.02 |  |  |  |  |
| **Icps1** | 40 | 488 | 121 | **Vcps1** | 80.2 | 76.9 | 6.17 |  |  |  |  |
| **Icps2** | 43 | 2460 | 573 | **Vcps2** | 55.1 | 289 | 15.9 |  |  |  |  |
| **Icps3** | 29 | 1610 | 561 | **Vcps3** | 60.9 | 37.5 | 2.28 |  |  |  |  |
| **Icps4** | 67 | 153 | 23.0 | **Vcps4** | 14.7 | 14.3 | 0.21 |  |  |  |  |
| **Icps5** | 59 | 304 | 51.7 | **Vcps5** | 66.2 | 43.2 | 2.86 |  |  |  |  |
| **Icps6** | 39 | 248 | 64.2 | **Vcps6** | 40.7 | 63.3 | 2.58 |  |  |  |  |
| **Icps7** | 60 | 255 | 42.9 | **Vcps7** | 85.2 | 52.2 | 4.45 |  |  |  |  |
| **Icps8** | 46 | 839 | 183 | **Vcps8** | 18.5 | 111 | 2.06 |  |  |  |  |
| **Icps9** | 42 | 1720 | 411 | **Vcps9** | 75.8 | 224 | 17.0 |  |  |  |  |
| **Icps10** | 42 | 6.9 | 1.70 | **Vcps10** | 76.2 | 0.3 | 0.02 |  |  |  |  |
| **(continuation of Table K)** | | | | | | | | | | | |
| **MinPaX II** | | | | **MinPaX III** | | | | **MinPaX IV** | | | |
| **Sample ID** | **weight sample [mg]** | **Se in digest [µg L-1]** | **Se in solid [ppm]** | **Sample ID** | **weight sample [mg]** | **Se in digest [µg L-1]** | **Se in solid [ppm]** | **Sample ID** | **weight sample [mg]** | **Se in extract [µg L-1]** | **Se in solid [ppm]** |
| **IIpac1** |  | 95.1 |  | **IIIpac1** |  | 0.6 |  | **IVpac1** |  | 3.9 |  |
| **IIpac2** |  | 5.1 |  | **IIIpac2** |  | 9.5 |  | **IVpac2** |  | 123 |  |
| **IIpac3** |  | 3.4 |  | **IIIpac3** |  | 121 |  | **IVpac3** |  | 1000 |  |
| **IIpac4** |  | 0.9 |  | **IIIpac4** |  | 2.6 |  | **IVpac4** |  | 59.0 |  |
| **IIpac5** |  | 49.4 |  | **IIIpac5** |  | 90.3 |  | **IVpac5** |  | 364 |  |
| **IIpac6** |  | 124 |  | **IIIpac6** |  | 45.6 |  | **IVpac6** |  | 977 |  |
| **IIpac7** |  | 9.2 |  | **IIIpac7** |  | 2.2 |  | **IVpac7** |  | 26.9 |  |
| **IIpac8** |  | 30.8 |  | **IIIpac8** |  | 17.7 |  | **IVpac8** |  | 145 |  |
| **IIpac9** |  | 51.6 |  | **IIIpac9** |  | 30.5 |  | **IVpac9** |  | 375 |  |
| **IIpac10** |  | 0.5 |  | **IIIpac10** |  | 0.9 |  | **IVpac10** |  | <0.1 |  |
| **IIcp1** | 71 | 2490 | 351 | **IIIcp1** | 106 | 404 | 38.1 | **IVcp1** | 110 | 72.8 | 8.01 |
| **IIcp2** | 97 | 268 | 27.6 | **IIIcp2** | 101 | 2230 | 221 | **IVcp2** | 72 | 445 | 32.0 |
| **IIcp3** | 98 | 2340 | 239 | **IIIcp3** | 81 | 1370 | 169 | **IVcp3** | 58 | 162 | 9.39 |
| **IIcp4** | 89 | 427 | 48.3 | **IIIcp4** | 105 | 403 | 38.2 | **IVcp4** | 90 | 43.7 | 3.93 |
| **IIcp5** | 88 | 821 | 93.8 | **IIIcp5** | 75 | 804 | 108 | **IVcp5** | 115 | 123 | 14.2 |
| **IIcp6** | 71 | 770 | 108 | **IIIcp6** | 95 | 1400 | 147 | **IVcp6** | 75 | 127 | 9.53 |
| **IIcp7** | 96 | 154 | 16.0 | **IIIcp7** | 83 | 285 | 34.3 | **IVcp7** | 107 | 57.0 | 6.10 |
| **IIcp8** | 70 | 1490 | 212 | **IIIcp8** | 88 | 1640 | 185 | **IVcp8** | 115 | 282 | 32.3 |
| **IIcp9** | 99 | 3170 | 321 | **IIIcp9** | 78 | 2620 | 336 | **IVcp9** | 116 | 403 | 46.7 |
| **IIcp10** | 104 | 3.0 | 0.30 | **IIIcp10** | 77 | 2.9 | 0.37 | **IVcp10** | 143 | 1.7 | 0.24 |
